# Inferring single-cell and spatial microRNA activity from transcriptomics data

**DOI:** 10.1038/s42003-025-07454-9

**Published:** 2025-01-18

**Authors:** Efrat Herbst, Yael Mandel-Gutfreund, Zohar Yakhini, Hadas Biran

**Affiliations:** 1 Arazi School of Computer Science, Reichman University, Herzliya, Israel; 2 Computer Science Department, Technion - Israel Institute of Technology, Haifa, Israel; 3 Faculty of Biology, Technion - Israel Institute of Technology, Haifa, Israel

**Keywords:** Statistical methods, Software, Computational models

## Abstract

The activity of miRNA varies across different cell populations and systems, as part of the mechanisms that distinguish cell types and roles in living organisms and in human health and disease. Typically, miRNA regulation drives changes in the composition and levels of protein-coding RNA and of lncRNA, with targets being down-regulated when miRNAs are active. The term “miRNA activity" is used to refer to this transcriptional effect of miRNAs. This study introduces miTEA-HiRes, a method designed to facilitate the evaluation of miRNA activity at high resolution. The method applies to single-cell transcriptomics, type-specific single-cell populations, and spatial transcriptomics data. By comparing different conditions, differential miRNA activity is inferred. For instance, miTEA-HiRes analysis of peripheral blood mononuclear cells comparing Multiple Sclerosis patients to control groups revealed differential activity of miR-20a-5p and others, consistent with the literature on miRNA underexpression in Multiple Sclerosis. We also show miR-519a-3p differential activity in specific cell populations.

## Abstract (web summary)

This study introduces miTEA-HiRes, a method designed to facilitate the evaluation of miRNA activity at high resolution. The method applies to single cell transcriptomics, type-specific single cell populations, and spatial transcriptomics data.

## Introduction

MicroRNAs (miRNAs) are small RNAs (sRNAs) that operate by post-transcriptionally repressing their target genes, either by inducing messenger RNA (mRNA) deadenylation and degradation or by inhibiting translation^1,2^. Many studies have shown that overexpression of miRNAs reduces the expression level of their targets^2–6^ and their protein levels^3,4,7^, while miRNAs knockdown elevates the expression levels of their targets^2,5,6^. miRNAs have a vital effect on many developmental and disease processes, making them potential theraputic targets in cancer^8–12^, cardiovascular disease^13–15^, neurodegenerative diseases^16^, diabetes mellitus^17^, achute ischemmic disease^18^, inflammatory disease^19^, depression^20^ and more^21–25^.

In the last twenty years, there has been significant progress in single-cell RNA-sequencing, resulting in data and insight for different cell types and across systems. This advancement has facilitated a thorough investigation of cell diversity in developmental and disease processes, relying almost exclusively on mRNAs and long non-coding RNAs (lncRNAs)^26,27^. The detection of miRNAs in single cells, however, is one step behind. Early attempts relied on PCR^28–32^, nanomotors^33^, atomic force microscopy^34^, isothermal amplification methods^35–37^, or FISH^38^, all of which are limited in the number of measured miRNAs. Small RNA single cell sequencing was first attempted by ref. ^39^. Then, other methods emerged for the simultaneous sequencing of sRNAs and mRNAs^40–43^. However, these are very limited in the number of cells profiled. The concept of adding polyA tails to nonpolyadenylated RNA strands, which was first introduced by ref. ^44^ for sequencing of small (<150 bp) DNAs or RNAs, has later matured into the methods now known as “totalRNA single cell sequencing". This approach detects RNA strands of all lengths by adding polyA tails to nonpolyadenylated RNA, either before or after fragementation^45–47^.

In the past decade, high-throughput spatial transcriptomics methods were developed^48^. Through spatially resolved gene expression, we can now not only identify biological processes in a tissue, but also pinpoint these processes to an exact location of the tissue^48–52^. Traditionally, these methods focused entirely on mRNAs and lncRNAs. However, very recently the STRS (spatial total RNA-sequencing) method was introduced^53^. STRS employs the same polyadenylation concept (mentioned earlier for single cell) to slices of tissue, resulting in a gene expression spatial map for genes of all lengths.

The efforts to measure miRNA expression in single-cell and spatially are important and would probably soon mature and become widespread. However, they do not immediately solve the open question of expression versus activity of miRNAs in a natural setting. The relationship between miRNA expression and its level of activity, that is- the extent of target repression, has been extensively researched^1,7,42,54^. Reference ^7^ found that regulation by miRNAs is non-linear: miRNA can behave both as a switch of target expression and as a fine-tuner. Reference ^12^ report on studying both miRNA and mRNA profiles in a cohort of breast cancer patients. The analysis leads to identifying miR29’s role in regulating the extra-cellular matrix, as well as other findings. Reference ^42^ found that in a few examples, activity depends on expression levels of both the miRNA and its targets, that is: miRNAs that were overall expressed had a negative correlation with their overall expressed targets. We also know that the relationship between a miRNA and its targets may be more complex than a simple negative regulation, and includes feedback loops, feed-forward loops and miRNA ’sponging’^54^. The recent emergence of totalRNA single-cell and spatial methods enables us to initiate an exploration of miRNA to target relationships at a higher resolution. However, current transcriptomics technology also allows for inference and insight, as we show in this work.

Several methods have been suggested to facilitate the study of miRNA activity. Our group has formerly developed miTEA, a method to predict miRNA activity by examining the mutual statistical enrichment in two ranked lists - the list of expressed genes and the list of targets for any given (pivot) miRNA (the output of a target prediction algorithm or experiment)^55^. MIENTURNET^56^, DIANA-mirExTra2.0^57^ and enrichMiR^58^ offer a miRNA target enrichment analysis for a set of genes based on the hypergeometric test. DIANA-mirExTra2.0 and enrichMiR also accept differential expression analysis results as input, and enrichMiR also offers other statistical approaches. However, all of these methods and tools focus on activity prediction for a two-condition experiment, and do not support the more complex architecture of single cell RNA sequencing experiments.^59^ developed miReact, which is a method aimed at evaluating miRNA activity in single cell RNAseq datasets based on motif enrichment. They describe sound statistics for this context and provide a friendly software package. Their approach, however, does not utilize database (experimentally validated) information and does not address spatial transcriptomics. Also, it does not use the activity scores obtained for subsets of the data to find significant differences in miRNA activity between cell types or conditions. Olgun et al.^60^ developed miRSCAPE, a computational tool to infer miRNA expression in single cell data. They hypothesize that measuring miRNA expression can be performed more accurately, exploiting the complex direct as well as indirect regulatory links between miRNA and the expression of other mRNAs. miRSCAPE demonstrates impressive results. However, the accuracy of the results relies on finding and pre-training a model using a bulk dataset of the same cell type and condition as the inspected single-cell dataset, in order to capture the relevant regulatory links. This makes miRSCAPE results highly variable, depending on the availability and quality of such bulk dataset, affecting the generalizability, reproducibility, and usability of the tool. Finally, all of the above mentioned tools do not address spatial RNA sequencing.

In this work, we present **miTEA-HiRes**: a statistical approach to interpret the activity of miRNAs based on the expression pattern of their targets in single-cell RNAseq and spatial transcriptomics. The method creates the list of targets by filtering the miRTarBase database^61^. It then uses the multiple-sample setting to perform normalization of gene expression values, and finally applies the minimum HyperGeometric (mHG) test^62,63^ to compute the level of activity in each sample. We use this approach for two main purposes. First, to create, for the first time to the best of our knowledge, miRNA activity maps for scRNAseq and spatial datasets, enabling the exploration of miRNA heterogeneity (variability) across different cell types or across the geography of a sample. Second, to find differentially active miRNAs between cells in different conditions, for example, disease vs. normal. We evaluate our method using bulk data, and single-cell miRNA induction experiments. We investigate the relationship between miRNA expression and activity, as emerges from total RNA single-cell sequencing.

Our method and usage instructions are available at https://github.com/EfiHerbst31/miTEA-HiRes.

## Results

### The statistical method

miTEA-HiRes relies on a precomputed miRNA-target interaction set, which was curated from the miRTarBase database as in ref. ^63^: a miRNA-target interaction is included in the interaction set if it is functional, and has at least one strong experimental support or two weak ones. Statistical characteristics of the final list of miRNA-target interactions can be found in Supplementary Fig. 1.

The miTEA-HiRes analysis has two steps. At the first step, it computes the activity *p*-values, representing the level of activity of every evaluated miRNA in each of the cells or spots. At the second step, miTEA-HiRes combines the activity *p*-values for each miRNA to yield activity scores, indicating its biological significance. miTEA-HiRes also produces activity maps and histograms when appropriate.

#### First step: computing activity *p*-values

**Fig. 1:**
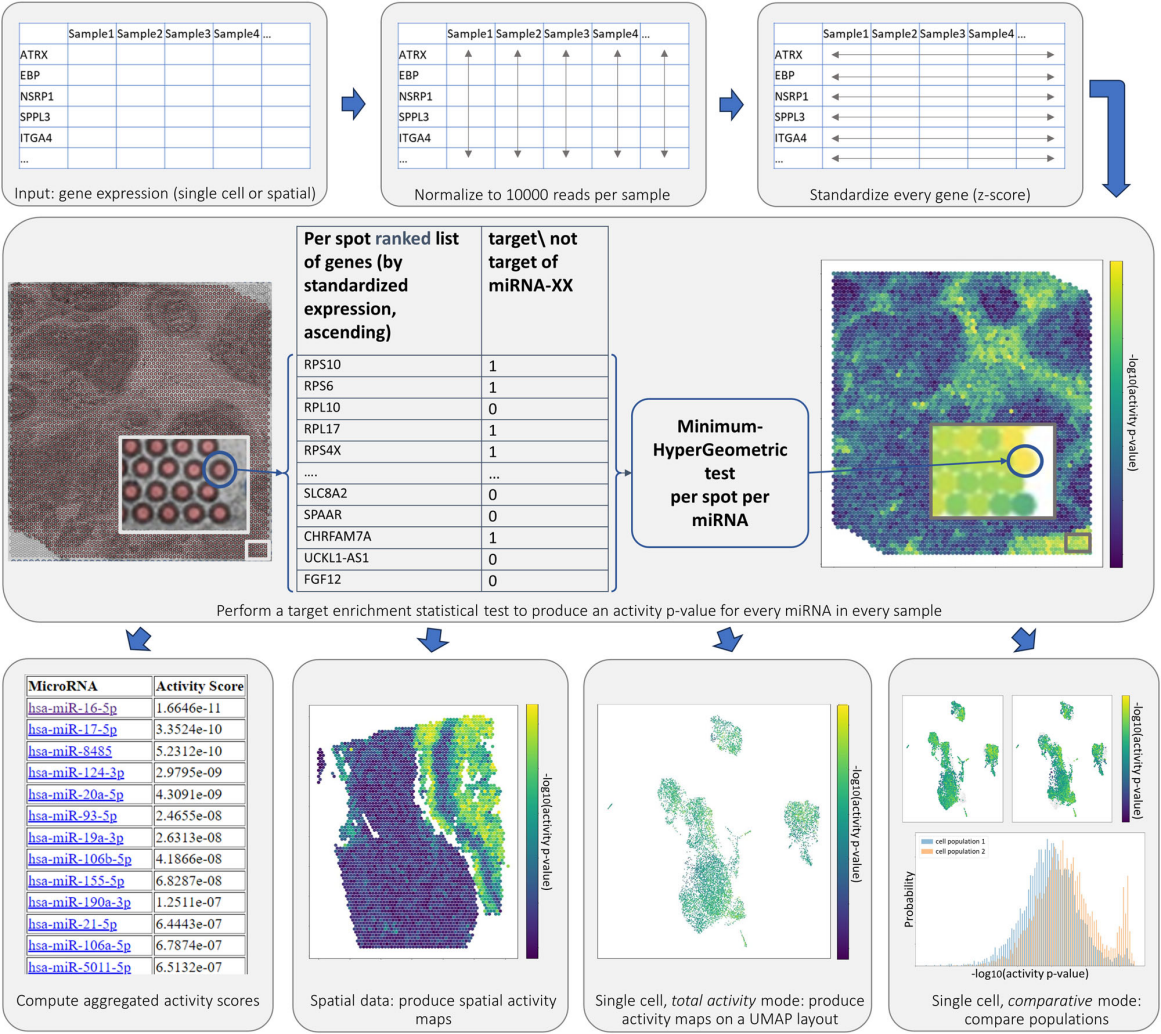
miTEA-HiRes pipeline. Top row: normalization and standardization. Middle row: computation of activity *p*-values (shown for spatial data. The same process applies to single-cell data). Bottom row: computation of aggregated activity scores (for all data types); Generation of spatial activity maps for spatial data, or activity maps on a UMAP layout for single-cell data; Statistical comparison between activity *p*-values of different cell populations for single-cell data in *comparative activity* mode. See text and Methods for more details.

As a preprocessing step (1, top row), miTEA-HiRes first performs normalization of total reads per spot (for spatial datasets) or cell (for single cell datasets). Next, it performs a Z-score transformation per gene. We recommend preprocessing data to account for batch effects^64^ if relevant, and to remove cells with very few reads (<1000) before using it as input for miTEA-HiRes, as these cells may disproportionately impact z-scores. Then, for each spot or cell, miTEA-HiRes ranks the genes in ascending order of their Z-scores (see Methods for the mathematical definitions). Our main assumption is that if a certain miRNA is active, then its targets would be down-regulated, so they would appear at the top of this ranked list (as depicted in Supplementary Fig. 3). Enriched occurrence is evaluated using the minimum-HyperGeometric (mHG) test^55,62,65–67^, a statistical procedure assessing the enrichment level of a set of elements at the top of a list. The mHG test is performed for each evaluated miRNA, for all spots\cells. The reported *p*-values of the mHG test are then used as the activity *p*-values associated with every pair of miRNA and cell\spot (Fig. 1, middle row). The activity matrix, containing the activity *p*-values, is provided as output for users to facilitate further user-defined analysis, in addition to the analysis performed by miTEA-HiRes pipeline. For example - an analysis based on clustering according to activity profiles can be performed. See Methods and pseudo-code (Supplementary Algorithm 1) for a more detailed description of this step.

#### Second step: computation of aggregated activity scores

**Fig. 2:**
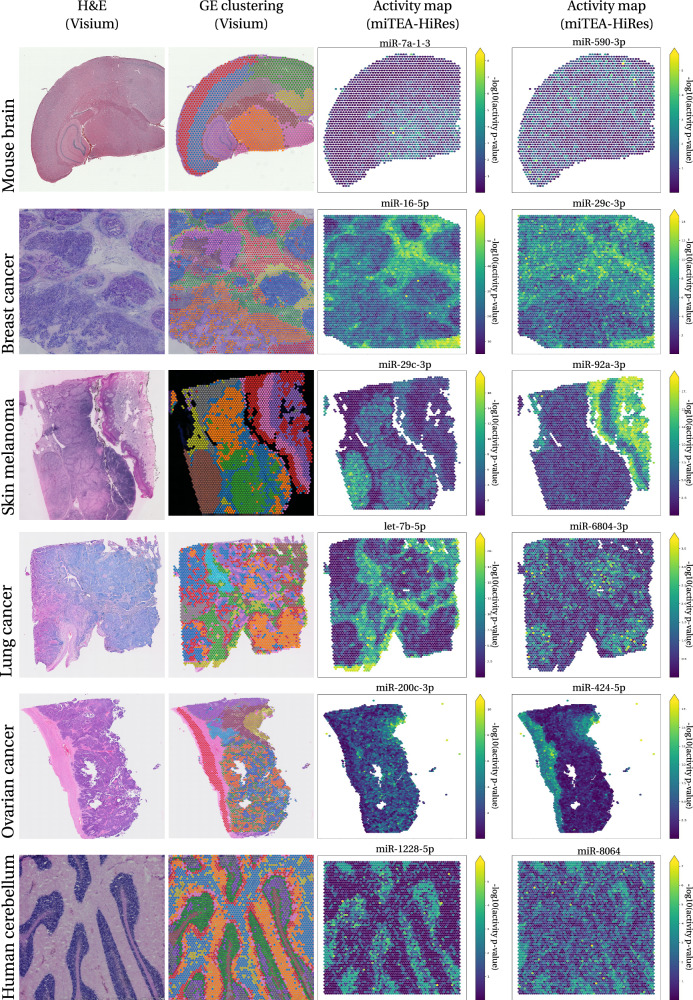
miTEA-HiRes spatial activity maps. Activity maps of two selected miRNAs per tissue are presented in the two rightmost columns. Colors of activity maps represent -log10 of activity *p*-values. Corresponding H&E slides and GE clustering, provided by Visium, appear in the two leftmost columns. Datasets presented (from top to bottom): mouse brain (2264 spots), human breast cancer (4898), human skin melanoma (3458), human lung cancer (3858), human ovarian cancer (4674) and human cerebellum (4992).

For spatial data, miTEA-HiRes computes the aggregated activity score of each miRNA as the percentage of active spots (defined by thresholding the activity *p*-values) and outputs a list of miRNAs sorted by this value. miTEA-HiRes also provides an activity map for each miRNA over the spatial coordinates (Fig. 1 bottom row and Fig. 2).

For single cell data, miTEA-HiRes has two working modes. The *total activity* mode treats the dataset as a single population of cells, so it does not require any cell annotations. In this mode, the aggregated activity scores are defined as the corrected average activity *p*-values (Methods). miTEA-HiRes then reports the miRNAs sorted by their activity scores, as well as an activity map for each miRNA over a UMAP layout (Fig. 1 bottom row).

**Fig. 3:**
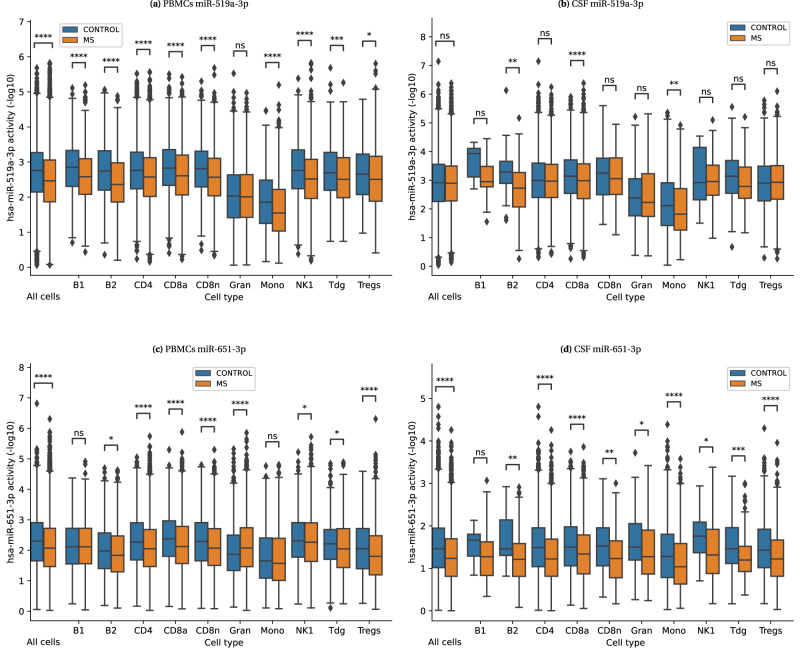
miRNA differential activity between MS and control groups at different resolutions. miRNA differential activity between Multiple Sclerosis (MS) and control at all-cells level (left most pair of boxes), compared to cell type specific level. **a** miR-519a-3p analysis on peripheral blood mononuclear cells (PBMCs); **b** miR-519a-3p in cerebrospinal fluid (CSF) cells; **c** miR-651-3p in PBMCs; **d** miR-651-3p in CSF cells. Two sided WRS test *p*-value legend: ns: *p* > 0.05; * : 0.01 < *p *≤ 0.05; ** : 1*e*−3 < *p* ≤ 0.01; *** : 1*e*−4 < *p *≤ 1*e*−3. **** : *p *≤ 1*e*−4. Cell-type keys: B1 and B2: B cell subsets; CD4: CD4+ T cells; CD8a: activated CD8+ T cells; CD8n: non-activated CD8+ T cells; Gran: granulocites; Mono: monocytes; NK1: natural killer cells; Tdg: *γ* *δ* T cells; Tregs: regulatory CD4+ T cells^77^. Group sizes can be found in Supplementary Table 4.

The second mode is the *comparative activity* mode. In this mode, miTEA-HiRes performs a two-sided Wilcoxon rank-sum (WRS) test to evaluate the difference in activity *p*-values between compared populations or conditions (for example: cell types, disease vs. control). miTEA-HiRes then defines the aggregated activity scores by correcting the WRS *p*-values using a false-discovery rate (FDR) correction. For this mode, the terms “activity score" and “corrected WRS *p*-value" are used interchangeably. miTEA-HiRes reports the miRNAs that have significant activity *p*-values in at least one of the compared populations and manifest significant differential activity between the populations (Methods). miTEA-HiRes also reports a histogram of the activity *p*-value distributions in the two populations (see Table 1, Fig. 1 bottom row). A difference in miRNA activity between two conditions reflects a difference in the expression values of its targets (See Fig. 3 and Supplementary Fig. 11).

**Table 1:** miTEA-HiRes analysis of MS single cell dataset of PBMCs

	*total activity* mode		*comparative activity* mode			
miRNA	rank	activity score	rank	activity score	Histogram of activity *p*-values	Literature
miR-106b-5p*	1	1.46e−16	1	3.03e−170		Increased MS-related disability is associated with down-regulation of miR-106b-5p and miR-19b-3p in RRMS patients^84^; belongs to the miR-106b/25 cluster^78^
miR-93-5p*	2	3.92e−15	2	3.19e-204		Regulates MS risk genes^81^; Found to be overexpressed in MS and in RRMS^81,82^; belongs to the miR-106b/25 cluster^78^
miR-17-5p*	3	6.50e−15	3	3.14e−165		Inhibits T Cell activation genes, and underexpressed in MS whole blood^134^; belongs to the miR-17/92 cluster^79^
miR-20a-5p	4	1.23e−14	5	1.58e−199		Inhibits T Cell activation genes, and underexpressed in MS whole blood^134^; belongs to the miR-17/92 cluster^79^
miR-16-5p*	5	1.90e−13	4	9.22e−140		Underexpressed in PBMCs, CD4+ T cells and B cells of RRMS patients, comparing to healthy subjects^135^
miR-519d-3p	6	1.35e−12	6	4.50e−158		No literature found
miR-19b-3p*	7	3.44e−12	7	4.50e−183		Increased MS-related disability is associated with downregulation of miR-106b-5p and miR-19b-3p in relapsing-remitting MS patients^84^; belongs to the miR-106a/363 cluster^80^
miR-20b-5p	8	3.49e−12	8	7.84e−152		Belongs to the miR-106a/363 cluster^80^, whose member miR-19b-3p is reported to be linked with MS^84^. The miR-106a/363 cluster is highly homologous to the miR-17/92 cluster, they are subsumed in miRBase as one family of miRNAs with similar target genes and functions^80,136,137^. The miR-17/92 cluster includes hsa-miR-17-5p and hsa-miR-20a-5p, which are known to be underexpressed in MS whole blood^134^.
miR-106a-5p*	9	8.80e−11	9	2.46e−162		Belongs to the miR-106a/363 cluster^80^, see cell above.
miR-19a-3p*	10	9.68e−10	>10	-	-	Belongs to the miR-17/92 cluster^79^
miR-155-5p	>10	-	10	3.07e−113		A crucial regulator of inflammation, modulates the autoimmune response in MS and affects the function of the brain-blood barrier in MS patients^138^; up-regulated in PBMCs of RRMS patients in the remission phase compared with healthy controls^83^

Top 10 most overall active miRNAs as computed by miTEA-HiRes in *total activity* mode, and top 10 differentially active miRNAs as computed by miTEA-HiRes in *comparative activity* mode: Multiple Sclerosis (MS) vs. control. In the histograms the x-axes indicate -log10(activity *p*-values). Histogram legend: Blue: MS, orange: control. miRNAs marked with * were ranked at the top 10 of the *total activity* mode for the MS single cell cerebrospinal fluid (CSF) dataset (Supplementary Table 1). Analysis in *comparative activity* mode resulted in identical top 10 lists of differentially active miRNAs for peripheral blood mononuclear cells (PBMCs) and CSF datasets (Supplementary Table 1). In *comparative activity* mode, miRNAs are ranked as described in Methods.

miTEA-HiRes thus takes single cell or spatial gene expression data (with or without cell annotations) as input, and outputs a list of miRNAs ranked by their potential biological interest, combining overall activity and differential activity, when relevant. miTEA-HiRes is available at Github: https://github.com/EfiHerbst31/miTEA-HiRes.

### miTEA-HiRes provides evidence of tissue-specific and localized patterns of miRNA activity, utilizing spatial transcriptomics

We downloaded spatial transcriptomics data of a mouse brain^68^, human breast cancer^69^, human skin melanoma^70^, human lung cancer^71^, human ovarian cancer^72^ and human cerebellum^73^ from the Visium website^74^. Fig. 2 shows the histology slides (H&E slides) along with gene expression (GE) clustering provided on the Visium website, and specific noteworthy miRNA activity maps exemplifying miTEA-HiRes’s output.

Some activity maps clearly resemble the H&E or the GE clustering- indicating miRNA activity in specific tissue types. For instance, miR-16-5p’s activity throughout the primary human breast cancer tissue is similar to its H&E slide (Fig. 2, second row). miR-16-5p is known to suppress breast cancer proliferation by targeting *ANLN*^75^, which is under-expressed in this dataset compared to other datasets (Supplementary Fig. 2). Another example is let-7b-5p, with an activity pattern that is similar to the GE clustering in the lung cancer slide (Fig. 2, fourth row). In the human cerebellum, the relatively understudied miR-1228-5p and miR-8064 seem clearly more active in specific regions that are also visible in the H&E slide and GE clustering (2, bottom row).

Other activity maps may offer a more unique insight- information about the tissue that cannot be inferred from the H&E or the GE clustering. For example, the activity of miR-29c-3p in the breast cancer sample (Fig. 2, second row) does not precisely correspond, at the qualitative visual inspection level, to either the clustering patterns or the characteristics observed in the H&E slide (Supplementary Fig. 10). In order to investigate whether the activity pattern might be affected by the sparse nature of spatial gene expression datasets, we experimented with downsampling the counts matrix (see Supplementary Fig. 12). miR-29c-3p is known to be a tumor suppressor miRNA^76^. Also, previous bulk analysis study showed that miR-29c is over-expressed in luminal A breast cancer compared to basal breast cancer, and that its targets tend to have lower expression values in breast cancer samples with elevated miR-29c expression, indicating that it is not only expressed but also active^12^. MiR-29 is known to play a role in extra-cellular matrix formation^12^ and in cardiovascular disease pathogenesis in mice^14^.

We found that some miRNAs are highly active only in certain tissue regions (for example, miR-424-5p in the ovarian cancer sample, 2, fifth row), while other miRNAs have a more dispersed activity pattern (miR-200c-3p, also in the ovarian cancer sample, Fig. 2, fifth row). Another interesting observation is that some miRNAs exhibit activity patterns that are disjoint from those of other miRNAs. For example, in the lung cancer tissue (Fig. 2, fourth row), the well-studied let-7b-5p and the less understood miR-6804-3p demonstrate non-overlapping patterns of activity. To quantify this observation, we compared the top 10% most active spots in let-7b-5p with those of miR-6804-3p and found zero overlap (hypergeometric *p*-value for under-enrichment 2*e*−19). When considering the top 20%, there was an overlap of only 8 spots (hypergeometric *p*-value for under-enrichment 8*e*−71).

It is important to note that not all miRNAs exhibit high activity, which is expected. In the evaluated samples, the fraction of miRNAs that were found to be active (-log10(*p*-value) > 5) in at least 20% of the spots was between 0% (in the mouse brain and human brain) and 10% (in the human breast cancer).

### miTEA-HiRes identifies disease-associated miRNAs at different resolutions in single-cell PBMCs and CSF datasets of Multiple Sclerosis patients

miTEA-HiRes was applied to a single-cell RNAseq dataset of peripheral blood mononuclear cells (PBMCs) obtained from five patients with Multiple Sclerosis (MS) and five control cases^77^(Table 1). miTEA-HiRes was also applied to a dataset of RNAseq of cerebrospinal fluid samples (CSF) collected from MS patients and a control group (Supplementary Table 1). The PBMCs dataset consisted of 42,969 cells and the CSF dataset consisted of 22,357 cells. The breakdown into cell type-specific groups can be found in Supplementary Table 4.

#### Comparing miRNA activity in MS to control

To reduce computation time, 10,000 cells were randomly selected from the PBMCs dataset (5000 from each group of MS and control). We then conducted the analysis using two modes: the *total activity* mode and the *comparative activity* mode - with the MS cell population vs. control.

We present and discuss miTEA-HiRes’s results by observing the 10 miRNAs ranked highest for overall activity (in *total activity* mode, without cell annotations), and the 10 miRNAs ranked highest for differential activity between MS and control (in *comparative activity* mode) (Table 1). For convenience, we refer to the first list as the *total activity* list and to the second list as the *comparative activity* list. The following miRNAs, which appear in both lists, have known connections to MS: miR-106b-5p and miR-93-5p (which belong to cluster 106b/25^78^), miR-17-5p and miR-20a-5p (which belong to the miR-17/92 cluster^79^), miR-19b-3p (which belongs to miR-106a/363^80^), and miR-16-5p. miR-155-5p, which only appears in the *comparative activity* list, also has a known connection to MS. Literature suggests that miR-106b-5p, miR-17-5p, miR-20a-5p, miR-16-5p, and miR-19b-3p are underexpressed or underregulated in MS patients compared to a control group (Table 1). This underexpression pattern fits the pattern of miRNA inactivity in MS cells depicted in miTEA-HiRes’s histograms (see Table 1). For two other miRNAs: miR-93-5p and miR-155-5p, whose miTEA-HiRes histograms also show an under-activity pattern in MS, literature suggests that they are over-expressed in MS (but not necessarily over-active) compared to healthy subjects^81–83^. We suggest that these miRNAs might have a complex role in MS that should be further investigated. The relationship between miRNA activity and expression is further explored in  the validation section.

miTEA-HiRes also detected some miRNAs which are not yet reported to be related to MS. Some of them, however, belong to the same or similar clusters as known miRNAs related to MS. For example: hsa-miR-20b-5p (*total activity* list # 8) and miR-106a-5p (*total activity* list # 9), which belong to the miR-106a/363 cluster (Supplementary Table 1,^80,84^).

Some of the highly ranked miRNAs are not well studied for their functionality, such as miR-519d-3p (Table 1). Following these results, miR-519d-3p could potentially be investigated for its relation to MS.

We found that nine miRNAs appeared in both lists. Also, all miRNAs in the *total activity* list were found to be differentially active between MS and control in *comparative activity* mode (corrected WRS *p*-values  < 9*e*−140). We further touch on this point in the discussion.

#### Cell type specific miRNA differential activity

We downloaded the PBMCs dataset with cell types. In order to improve statistical significance when analyzing cell subsets, we considered the entire dataset for this section, without sampling. We then applied miTEA-HiRes to the data in *comparative activity* mode, to compare the activity of miRNAs between MS and control within each cell type (Table 2, Supplementary Table 2), and also to compare miRNAs activity between cell types within the MS or control groups (Table 3, Supplementary Table 3). Full Results can be found at Zenodo 10.5281/zenodo.10720979.

**Table 2:** Comparison between MS and control within a specific cell-type

Cell type and origin	miR-19b-3p	miR-519a-3p	miR-3609	miR-651-3p
non-activated CD8+ T cells, PBMCs	1.65e−26 *↓*	1.52e−13 *↓*	5.52e−11 *↓*	1.20e−06 *↓*
activated CD8+ T cells, PBMCs	1.02e−76 *↓*	4.89e−21 *↓*	9.63e−66 *↓*	8.49e−16 *↓*
CD4+ T cells, PBMCs	3.36e−105 *↓*	1.28e−40 *↓*	8.27e−75 *↓*	4.01e−38 *↓*
regulatory CD4+ T cells, PBMCs	1.51e−07 *↓*	2.76e−02	2.39e−13 *↓*	9.20e−05 *↓*
natural killer cells, PBMCs	3.77e−25 *↓*	6.74e−16 *↓*	6.17e−24 *↓*	5.29e−02
B1 (B cell subset), PBMCs	9.46e−13 *↓*	2.61e−14 *↓*	1.70e−05 *↓*	8.91e−01
B2 (B cell subset), PBMCs	7.92e−10 *↓*	1.47e−08 *↓*	3.05e−05 *↓*	7.06e−02
granulocytes, PBMCs	1.62e−05 *↓*	8.24e−01	3.76e−06 *↓*	3.67e−13 *↑*
activated CD8+ T cells, CSF	5.72e−14 *↓*	6.75e−08 *↓*	2.12e−03 *↓*	2.07e−19 *↓*
CD4+ T cells, CSF	4.68e−21 *↓*	2.14e−01	2.84e−01	1.45e−84 *↓*
regulatory CD4+ T cells, CSF	5.03e−02	6.22e−01	4.48e−01	4.45e−10 *↓*

For each cell type, we compared the Multiple Sclerosis (MS) cell population to the control population in *comparative activity* mode. The table lists corrected WRS *p*-values for miR-19b-3p, miR-519a-3p, miR-3609 and miR-651-3p, for each comparison. *↓*, *↑* represent reduced\elevated activity in MS, respectively. Activity was reduced in MS compared to control in all significant instances (*p*-values < 0.01) except one: miR-651-3p in granulocytes, where activity in MS was elevated compared to control. Group sizes can be found in Supplementary Table 4. More results can be found in Supplementary Table 2. Full results can be found at: 10.5281/zenodo.10720979.

**Table 3:** Comparison between CD4+ T cells and activated CD8+ T cells in the MS PBMCs and CSF

CD4+ T cells vs. activated CD8+ T cells	miR-19b-3p	miR-519a-3p	miR-3609	miR-651-3p
PBMCs control	3.38e−01	6.99e−04 *↑*	3.66e−02	4.89e−05 *↑*
PBMCs MS	3.73e−17 *↓*	4.48e−02	5.90e−09 *↓*	2.10e−06 *↑*
CSF control	1.27e−01	1.05e−10 *↑*	1.31e−04 *↑*	1.76e−01
CSF MS	7.87e−03 *↓*	9.94e−01	2.68e−01	6.84e−06 *↑*

miRNAs are the same as shown in Table 2. Numbers represent the corrected WRS *p*-values associated with this differential activity comparison. More results can be found in Supplementary Table 3. *↓*, *↑* represent reduced\elevated activity in activated CD8+ T cells compared to CD4+ T cells, respectively. Group sizes can be found in Supplementary Table 4.

#### Comparison between MS and control groups within specific cell types

We compared miRNA activity *p*-values between MS and control within the same cell type, in the *comparative activity* mode. Table 2 shows the comparison results for four selected miRNAs. Full results can be found at Zenodo 10.5281/zenodo.10720979. This comparison allows us to further explore the origin of differential activity seen in non-cell-type specific analyses (Fig. 3, Table 1, Supplementary Table 1). For example, miR-519a-3p was found to have differential activity between PBMCs obtained from MS patients and control (Table 1, corrected WRS *p*-value: 4.50*e* − 158). However, following the cell type-specific comparison, we can determine that this difference is subtle or absent in granulocytes and regulatory CD4+ T cells, but very prominent in other lymphocytes (Fig. 3, Table 2). On the other hand, mir-519a-3p was not found to be differentially active between CSF cells obtained from MS patients and control, and cell-type specific comparison reveals differential activity in activated CD8 cells (Fig. 3). A noteworthy observation is that both miR-519a-3p and miR-3609 are significantly differentially active between MS and control in PBMCs CD4+ cells, but not in CSF CD4+ cells (Table 2). This is an interesting finding considering the role these cells play in MS^85^. We note that we could not find literature elucidating the roles of miR-519a-3p and miR-3609 in MS.

Another example is miR-651-3p, which was also found to have differential activity between MS and control when considering all PBMCs (though it was not ranked at the top 10; corrected WRS *p*-value: 3.62*e*−97) and also when considering all CSF cells (corrected WRS *p*-value: 5.96*e*−96). By observing the cell-type specific comparisons (Fig. 3 and Table 2), we can conclude that this difference originates from T cells (both in PBMCs and CSF), but is much less prominent in natural killer cells. Interestingly, granulocytes in PBMCs demonstrate a significant opposite activity pattern with the MS group more active than the control group (Fig. 3).

The variation in miRNA activity values between conditions stems from differences in the expression of their target genes. For example, miR-519-3p shows reduced activity in B-cells from MS patients’ PBMCs compared to controls (Fig. 3), suggesting that its targets may be less down-regulated (more expressed) in MS compared to controls. To test this, we conducted the comparisons as in Fig.  3, but compared target expression between cell groups (rather than activity) (see Supplementary Fig. 11). In all cases where activity differences were significant, we observed a corresponding significant change in target expression (zero false positives). In 7 out of 44 comparisons, differences in target expression were significant but no significant changes in activity were captured.

#### Comparison between cell types within MS or control cell populations

Table 3 describes one example for comparison between different cell types: CD4+ T cells (CD4) and activated CD8+ T cells (CD8a). Notably, miR-19b-3p is significantly more active in CD4 cells compared to CD8a cells derived from PBMCs of MS patients, and also (though less significantly) in CSF derived from MS patients.

The case is different for miR-519a-3p, which has a significant difference in activity levels between CD4 and CD8 cells in both control PBMCs and CSF, but this difference, in both sample populations, is lost in MS patients.

More results can be found in Supplementary Table 3. Full results can be found at Zenodo 10.5281/zenodo.10720979.

We note that while the presented WRS *p*-values are corrected for the number of evaluated miRNAs, they are not adjusted for multiple pairwise comparisons, for instance, between all pairs of cell types. Users can correct their *p*-values for multiple comparisons by multiplying by the number of comparisons. For the MS dataset, even if all relevant pairwise comparisons are performed (~100), this correction would not affect conclusions regarding significant patterns (Tables 2 and 3).

### miTEA-HiRes identifies unique differentially active miRNAs with potential association to breast cancer metastasis

**Fig. 4:**
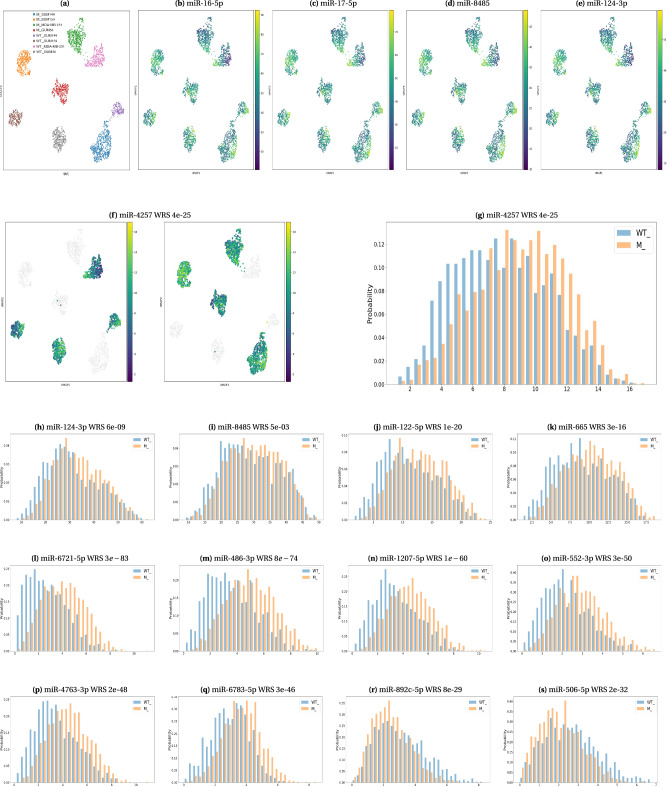
miRNA activity layouts for migratory and static breast cancer cells. **a** UMAP layout of the data. Colors represent cell-line and status. “WT_" refers to static cells, “M_" refers to migratory cells. **b**–**e** Activity maps in a UMAP layout, as produced by miTEA-HiRes in *total activity* mode. Colors depict -log10(activity *p*-values). **f** Comparative activity map in a UMAP layout, as produced by miTEA-HiRes in *comparative activity* mode. Left: static population, right: migratory population. Colors depict -log10(activity *p*-values). **g**–**s** Histograms of activity p-values divided by populations, as produced by miTEA-HiRes in *comparative activity* mode. In the histograms the x-axes indicate -log10(activity *p*-values). “WT_" refers to static cells, “M_" refers to migratory cells. More results are available in Supplementary Fig. 4. Full results are available at 10.5281/zenodo.10720979.

We analyzed single-cell RNAseq data from three breast cancer cell lines (MDA-MB-231, SUM149, SUM159) and from patient-derived cells (GUM36)^86^. As described in ref. ^86^, the samples were enriched for migratory breast cancer cells, and the migratory cell population was microfluidically separated from static cells (Fig. 4). The authors aimed to identify genes associated with cell migration and clinical outcomes in breast cancer, which could potentially serve as prognostic biomarkers. In line with this research direction, we sought to explore whether differentially active miRNAs could further improve our understanding of the process and possibly also serve as prognostic biomarkers in breast cancer and specifically as markers of metastasis.

To enhance the statistical power and leverage the similarities between breast cancer samples, we merged all cell lines and patient-derived cells into two groups: 1182 static cells and 1992 migratory cells. We then used the miTEA-HiRes analysis pipeline to identify both overall active miRNAs in breast cancer (*total activity* mode), as well as miRNAs uniquely active (or uniquely inactive) in migratory cells (*comparative activity* mode). Full results are available at Zenodo.

Upon applying miTEA-HiRes in *total activity* mode, we found that the top 10 active miRNAs are all known oncogenic miRNAs: miR-16-5p^11^, miR-17-5p^11^, miR-8485^87^, miR-124-3p^88^, miR-20a-5p^11^, miR-93-5p^12^, miR-19a-3p^11^, miR-106b-5p^89^, miR-155-5p^11^ and miR-190a-3p^90^. As seen in the activity maps of miR-16-5p, miR-17-5p, miR-8485 and miR-124-3p (Fig. 4), these miRNAs are universally active (activity *p*-values for all cells  < 1*e*−8, representing activity in all cells); also, their activity patterns are similar to one another (see Discussion for more comments on these findings). Next, we turned to *comparative activity* mode to find miRNAs that are differentially active between the static and the migratory populations. Out of the top 10 miRNAs in *total activity* mode, only two of them: miR-124-3p and miR-8485 were also found to be differentially active between the static and the migratory populations (*comparative activity* mode, corrected WRS *p*-values: 6*e*−09 and 5*e*−03 respectively). Their histograms of activity values are shown in Fig. 4. miR-124-3p is known to promote proliferation^88^ and metastasis^91^ of triple-negative breast cancer. Since the three breast cancer cell lines are from triple-negative origin, it is likely that miR-124-3p has a similar role here. Not much is known about miR-8485 in the context of breast cancer. However, it was found to promote proliferation and migration in mesenchymal stem cells derived from oral carcinoma^87^. The other eight miRNAs in the list of top 10 miRNAs in *total activity* mode had no significant differential activity between migratory and static populations (*comparative activity* mode, corrected WRS *p*-values  > 0.01).

Our analysis yielded additional miRNAs observed to be significantly differentially active between migratory and static cells. We found five miRNAs that were ranked at the top 30 in *comparative activity* mode (See Methods for details) and also had corrected WRS *p*-value  < 1*e*−15: miR-122-5p (1*e*−20, Fig. 4 and Supplementary Fig. 4e), miR-665 (3*e*−16, Fig. 4 and Supplementary Fig. 4f), miR-125b-5p (4*e*−19, Supplementary Fig. 4a and 4c), miR-125a-5p (5*e*−17, Supplementary Fig. 4b and 4d) and miR-4257 (Fig. 4). All of which were more active in migratory cells compared to static cells. miR-122-5p has been previously identified as a promoter of aggression and epithelial-mesenchymal transition in triple-negative breast cancer^92^. miR-665 is associated with metastasis and poor survival in breast cancer patients^93^. The differential and overall increased activity of miR-4257 is an interesting finding. While there is no specific indication of its involvement in breast cancer metastasis, the literature indicates inflammation-associated miR-4257 as a promoter of malignancy in colorectal cancer^94^. An additional study found upregulated exosomal miR-4257 in non-small cell lung cancer patients with recurrence compared with those without recurrence^95^. Considering these findings, miR-4257 may be further investigated to determine its role in breast cancer progression. As to miR-125b-5p and miR-125a-5p, there is contradictory evidence for these two miRNAs, suggesting that they inhibit the metastatic potential of breast cancer cells^96,97^. Further investigation is required in a dedicated study to explore this inconsistency, as there could be a more complex mechanism explaining this relationship between expression and activity in this case.

Other miRNAs were not ranked at the top 30 of *comparative activity* mode (Methods), however they show highly significant differential activity (corrected WRS *p*-value  < 1*e*−40) and substantial activity in at least one of the populations (activity *p*-values below 1*e* − 4 among at least third of cells in at least one population). These include miR-6721-5p (corrected WRS *p*-value: 3*e*−83, Fig. 4 and Supplementary Fig. 4g), miR-486-3p (8*e*−74, Fig. 4 and Supplementary Fig. 4h), miR-1207-5p (1*e*−60, Fig. 4 and Supplementary Fig. 4i), miR-552-3p (2*e*−50, Fig. 4 and Supplementary Fig. 4j), miR-4763-3p (2*e*−48, Fig. 4 and Supplementary Fig. 4k) and miR-6783-5p (2*e*−46, Fig. 4 and Supplementary Fig. 4l). These miRNAs are more active in migratory cells when compared to static cells. Both miR-486-3p and its opposite strand miR-486-5p are known to have central roles in several types of cancer, including functions relating to metastasis and cell migration^11,98^ (though we could not find any evidence specific to breast cancer). Regarding miR-1207-5p, the literature suggests that it promotes breast cancer cell growth by targeting *STAT6*^99^. miR-552 may also contribute to cell proliferation and migration^100^. The other microRNAs in this list have not been extensively studied in the context of cancer metastasis.

Two examples of miRNAs that are less active in migratory cells are miR-892c-5p (8*e*−29, Fig. 4 and Supplementary Fig. 4m) and miR-506-5p (2*e*−32, Fig. 4 and Supplementary Fig. 4n). For both of these miRNAs, their activity maps reveal that the difference in activity levels is most prominently observed in the primary tissue, wherein the static population has elevated activity comparing to the migratory population. The specific role of these miRNAs in relation to metastasis in breast cancer remains unknown. However, it is worth noting that miR-506-5p has been reported to be sponged by *FOXD2-AS1*, thereby having a role in glioma metastasis^101^. This finding could potentially explain the reduced activity observed in migratory cells compared to static cells.

Some miRNAs have very similar activity maps. One set is the pair miR-125a-5p (Supplementary Fig. 4d) and miR-125b-5p (Supplementary Fig. 4c), which belong to the miR-10 family^102^. In this case, the similarity of their activity maps can be explained by the fact that their mature sequence similarity is high (0.682^102^) and that their target lists greatly overlap (hypergeometric *p*-value 5.42*e*−62). Another pair of miRNAs, miR-892c-5p (Supplementary Fig. 4m) and miR-506-5p (Supplementary Fig. 4n), also have somewhat similar activity maps. They do not belong to the same family^102^, however they too are similar in sequence (mature sequence similarity 0.762^102^) and in target lists (hypergeometric *p*-value 1.63*e*−29). Some similarity also exists between miR-486-3p (Supplementary Fig. 4h), miR-1207-5p (Supplementary Fig. 4i), miR-6721-5p (Supplementary Fig. 4g) and miR-4763-3p (Supplementary Fig. 4k). They also have somewhat similar mature sequences (≥0.238 for all pairwise comparisons^102^) and similar target lists (hypergeometric *p*-value  < 1.8*e*−9 for all pairwise comparisons).

miR-16-5p, miR-17-5p, miR-8485, and miR-124-3p (Fig. 4) present an interesting case. While the ranges of activity *p*-values differ from one miRNA to another, their overall pattern is almost identical. These miRNAs have no significant overlap of their target lists (hypergeometric *p*-value  > 0.26 for all pairwise comparisons). Also, the mature sequence similarity between them is low (≤0.273 for all pairwise comparisons of miR-16-5p, miR-17-5p, and miR-124-3p; data of the sequence similarity of miR-8485 were missing from miRPathDB). To investigate whether the low expression targets of these miRNAs are involved in similar biological processes, we conducted pathway enrichment analysis using the canonical pathways gene set collection of the Human Molecular Signatures Database (MSigDB)^103–105^. Our analysis revealed 10 pathways enriched (hypergeometric *p*-value ≤0.01) among the low expression targets of miR-16-5p, miR-17-5p and miR-124-3p (Supplementary Fig. 5), almost all of them related to cancer. Further research is required to elucidate their specific functions, as well as the potential involvement of miR-8485.

To allow users to easily determine whether the target lists of two miRNAs greatly overlap, we computed the Jaccard index for every pair of miRNA target lists. The resulting table is available at Zenodo 10.5281/zenodo.10720979.

### Validation and comparison to other methods

#### Validation on bulk data from the NCI Genomic Data Commons (GDC) database and comparison to miREACT

We investigate the relationship between miTEA-HiRes activity *p*-values and measured expression by using data from the GDC portal^106^, which includes cancer tumors with matched bulk RNAseq and bulk miRNAseq samples. Similarly, we compare miReact^59^ activity scores to measured expression.

**Fig. 5:**
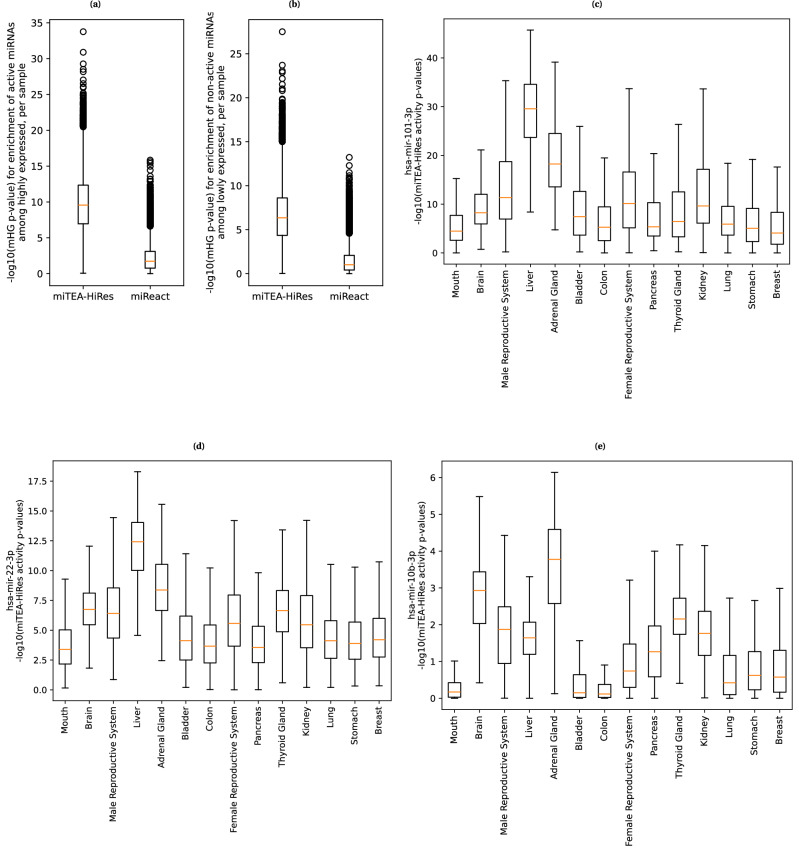
miTEA-HiRes validation and comparison to miReact, using the GDC bulk dataset. Agreement between expression and activity, per sample, reflected by the enrichment of: **a** active miRNAs among the most expressed miRNAs; **b** non-active miRNAs among the least expressed miRNAs; miTEA-HiRes activity *p*-values grouped by cancer tissue for: **c** miR-101-3p; **d** miR-22-3p; **e** miR-10b-3p. Full results are available at Zenodo 10.5281/zenodo.10720979.

We established a list of 11,927 cases in the GDC portal that have both bulk RNAseq sample and a miRNAseq sample, from a solid tissue primary tumor (Methods under GDC data curation). The dataset included tumors in 140 tissue types. We ran miTEA-HiRes and miReact on the RNAseq counts to predict miRNA activity *p*-values (Methods under Evaluating agreement between activity and expression in the GDC dataset, for miTEA- HiRes and miReact). For each sample, we sorted the miRNAs by descending expression, and used the mHG test to evaluate whether active miRNAs are over-represented at the top of the list (Fig. 5). Similarly, we computed the over-representation of non-active miRNAs at the bottom of the list (Fig. 5). Briefly, active vs. non-active is determined by *p*-value < 0.05, *p*-value > 0.1, respectively, in both tools. As seen in Fig. 5, in both cases, the agreement between expression and activity was better for miTEA-HiRes than for miReact.

We also present anecdotal examples of miRNAs with known functions in specific cancers (Methods under Activity and expression across cancer tissues of origin). Their expression levels are, indeed, reflected in their activity scores. For example, miR-101-3p has elevated activity, as determined by miTEA-HiRes, in the liver and the adrenal gland, compared to other cancer types (Fig. 5). Literature suggests it has a significant role in hepatocellular cancer^107–109^, and also in adrenocortical carcinoma^110^. Similarly, miR-22-3p has a role in heptocellular cancer^111,112^ and in adrenocortical carcinoma^113^, as reflected in its activity levels (Fig. 5). And finally, miR-10b-3p has a role in adrecortical carcinoma^110^, as reflected in its activity levels, as inferred by miTEA-HiRes (Fig. 5). The expression data of these three miRNAs is depicted in Supplementary Fig. 6. Full results are available at Zenodo 10.5281/zenodo.10720979.

#### Validation using two single-cell total-RNA datasets showcases significant relationship between miRNA expression and activity

We further investigate the relationship between miRNA activity and expression by analyzing total-RNA sequencing data obtained using the Smart-seq-total method^45^. This method allows for the simultaneous measurement of single-cell mRNA and miRNA expression. The datasets consist of RNAome data from human primary fibroblasts, from HEK293T and MCF7 cells (which were treated as a single multi-cell type dataset consisting of 633 cells), as well as from induced murine embryonic stem cells differentiated into embryoid bodies (2167 cells)^45^.

We quantified the association between expression and activity by first sorting the miRNAs in each dataset by expression, then measuring the enrichment of active (non-active) miRNAs, as assessed by miTEA-HiRes (Methods under Evaluating the relationship between single-cell miRNA expression and activity), at the top (bottom) of the list. We utilized the mHG test to determine the level of enrichment. This process was performed for both human and mouse datasets (see Methods and enrichment plots in Supplementary Fig. 7). For the human dataset we found a significant enrichment of active miRNAs at the top of the list (mHG *p*-value  = 3.76*e*−05). We also found a significant enrichment of non-active miRNAs at the bottom of the list (*p*-value  = 0.0055). For the mouse dataset, significant enrichment (<0.05) was detected on both sides as well; enrichment of active miRNAs at the top of the list (*p*-value  = 0.0301) and non-active miRNAs at the bottom of the list (*p*-value  = 0.0114).

We also tested the agreement between single-cell expression and activity in a single-cell resolution, for specific miRNAs that were found to be both overall active and expressed. Supplementary Fig. 8 shows example results from this analysis.

We were hoping to explore the relationship between the expression and activity of miRNAs using additional total-RNA datasets generated by VASA-seq^46^ and STORM-seq^47^ technologies. However, we found that these technologies report very low expression values of miRNAs, probably due to low miRNA capture efficiency.

#### Validation using scRNA-seq from miRNA induction experiment

Inspired by miRSCAPE^60^ validation, we sought to find whether miTEA-HiRes is able to accurately capture the differential expression of miR-199a-5p and miR-199a-3p, induced in i199 and i199-KTN1 cell lines (derived from HEK293 cell line, by using a plasmid containing the pri-miRNA)^114^. Towards this, we obtained the count matrices of 3280 i199 cells, and 3143 i199-KTN1 cells, including induced and uninduced annotations directly from the author^114^. We applied miTEA-HiRes on the two datasets, and found that for both cell lines, both miR-199a-5p and miR-199a-3p were significantly more active in the induced cells compared to the uninduced ones (see Methods under Evaluating differential activity in single-cell miRNA induction experiment). Two-sided WRS *p*-values for induced vs. uninduced cells are as follows. For induced i199 (695 cells) vs. uninduced (1561 cells), we get *p*-value = 7*e*−15 for miR-199a-3p and *p*-value =1*e*−10 for miR-199a-5p. For induced i199-KTN1 (611 cells) vs. uninduced (1075 cells), we get *p*-value = 0.001 for miR-199a-3p and *p*-value = 0.007 for miR-199a-5p. As reported by miRSCAPE^60^ authors, miRSCAPE results were accurately aligned with the induction of both miRNAs while miReact could not consistently detect the upregulation of miR-199 in induced cells. Results are available at Zenodo 10.5281/zenodo.10720979.

## Discussion

We developed miTEA-HiRes, an algorithmic approach, implemented as a Python package (including a pip-installable version), to predict miRNA activity in single cell RNA-seq and spatial transcriptomics datasets. miTEA-HiRes relies on experimentally validated miRNA-target interactions and on the mHG statistics. We used miTEA-HiRes to predict miRNA activity in six spatial datasets by Visium (Fig. 2), single cell datasets of PBMCs and CSF from MS patients and a control group (Fig. 3, Tables 1–3, Supplementary Tables 1–3), as well as in a single cell dataset of stationary and metastatic cells of breast cancer cell lines and a primary tumor samples (Fig. 4 and Supplementary Fig. 4). We also evaluated the relationship between expression and activity of miRNAs using totalRNA data (Supplementary Figs. 7 and  8). Finally we compare our method to miREACT using bulk GDC data (Fig. 5), and to both miREACT and miRSCAPE using miRNA induction experiment in single-cell data.

By employing miTEA-HiRes on spatial transcriptomics data, it is possible to obtain information about the spatial distribution of miRNA activity, information that cannot be directly obtained from standard measurement. The inferred information then allows us to determine, for example, whether the miRNA activity is concentrated in one location or spread across the tissue, whether it coincides with specific tissue regions identified by H&E staining, by standard clustering, or by adjacent IHC, and if it overlaps with the activity patterns of other miRNAs within the same sample. For example, in the lung cancer sample, let-7b-5p and miR-6804-3p were found to have significantly non-overlapping activity maps which suggests a synthetic-lethality behavior for this pair of miRNAs. This finding provides insight into miR-6804-3p’s function by drawing on the established functions of let-7b-5p. Another example is the activity of the tumor suppressor miR-16-5p, which is located in a well-defined area of the breast cancer sample, an area that has a distinct H&E staining pattern and that is marked at the level of full gene expression clustering. This activity pattern stands in contrast with the activity of another tumor suppressor, miR-29c-3p, that is far more spread out across the same tissue (Fig. 2).

For single cell data, miTEA-HiRes has two optional modes: *total activity* mode, which reports on miRNAs that are overall active, and *comparative activity* mode, which compares between populations of cells. We found that in some scenarios, miRNAs that are generally more active in a sample (reported by the *total activity* mode) greatly overlap with miRNAs that are differentially active between the sample main populations (reported by the *comparative activity* mode). An example observation is found analyzing MS and control PBMCs and CSF cells. In this analysis, we see a significant share of miRNAs that were found to be overall active and to also be differentially active between the MS and control populations. In other scenarios, such as the stationary and metastatic breast cancer cells, distinct sets of miRNAs were reported by the different modes, that is- miRNAs that were found to be highly active in all cells, did not have a significant overlap with the ones that were found to be differentially active between the stationary and metastatic populations. The variation of this aspect between the two datasets could potentially be attributed to the unique nature of these pathologies and the varying roles that miRNAs play in their formative and developmental processes.

We found that in the breast cancer single cell dataset, some sets of miRNAs had very similar activity maps. The activity map similarity of most of these sets can be easily explained by the fact that their mature sequence similarity is high or by the (related) fact that their target gene lists greatly overlap. However, the set miR-16-5p, miR-17-5p, miR-8485 and miR-124-3p (Fig. 4) cannot be so simply explained. This, therefore, is an insight about a possible similarity of functions between these miRNAs (or their targets), or perhaps a biological process that involves all of them. A future extension of miTEA-HiRes may include this downstream analysis step of spotting these very interesting sets of possibly cooperating miRNAs. Currently, users can use the Jaccard index similarity table available at Zenodo 10.5281/zenodo.10720979 to evaluate the overlap between miRNA target lists.

In some cases, miTEA-HiRes found that miRNAs were universally active, that is, active in all (or almost all) cells or spots. Examples of this phenomenon are given by mir-106b-5p in the MS PBMCs data (Supplementary Fig. 9), and four miRNAs in the breast cancer dataset mentioned above (miR-16-5p, miR-17-5p, miR-8485, and miR-124-3p; Fig. 4). The phenomenon may be biologically explained by the fact that some miRNAs have multiple isomiRs: miRNA variants that originate from the same loci, but are somewhat different due to various processes such as RNA editing, SNPs, or imprecise cleavage^115,116^. While a miRNA may have a large number of targets, each specific isomiR is more inclined to target a subset of these targets. If different isomiRs are present at different regions of the tissue or at different cell populations, the subset of targets being down-regulated would change between cells\spots. However- the miRNA would be considered universally active, from our statistical perspective. Mathematically, this finding can be counter-intuitive due to the normalization step. However, the same logic applies here: different subsets of the target list are down-regulated in different subsets of the cells\spots. To address this mathematical phenomenon we present a synthetic count matrix (see Supplementary Fig. 9).

An important aspect of miTEA-HiRes is its ability to facilitate the comparison of miRNA activity between subpopulations of the data, allowing researchers to derive more precise conclusions. For example, miR-651-3p was found to be significantly differentially active between MS and control in both PBMCs and CSF cells. The activity scores indicated a decreased activity in MS compared to the control group overall. However, only when comparing each cell type separately, we found that this pattern predominantly manifests in T cells. Surprisingly, in PBMCs, granulocytes exhibit an opposite pattern, with miR-651-3p being significantly more active in MS compared to the control group (Fig. 3, Table 2). As demonstrated by comparing Fig. 3 and Supplementary Fig. 11, significant differences in miRNA activity between conditions consistently reflect changes in the expression levels of their targets. The ability to compare subpopulations also effectively addresses another concern. When analyzing the two primary populations within the data, their composition can influence the outcomes. For instance, if a specific cell type is more prevalent in the first population and has higher activity of a particular miRNA, comparing the populations as a whole would simply indicate that the first group is more active for this miRNA. However, conducting comparisons among cell types within each group, as well as between the groups, would provide a better understanding of the findings.

The connection between the expression level of a miRNA and its activity level is an open question, not yet well addressed due to scarce resources that offered measurement of both. We explored this connection using a total RNA mouse and human datasets. The datasets include both the expression levels of mRNAs and lncRNAs (miRNA targets, which can be used by miTEA-HiRes to predict activity levels), and the expression levels of the miRNAs themselves. In both datasets, we found a significant enrichment of active miRNAs among highly expressed miRNAs (Supplementary Fig. 7), and also a significant enrichment of non-active miRNAs among low expression miRNAs. This finding is even more remarkable given that these datasets include the expression of the 3p and 5p strands of each miRNA combined, while they appear separately in the miRNA-target database thus yielding possibly different miTEA-HiRes results.

We also examined the relationship between expression and activity in MS, by scanning the literature for what is known about the expression of miRNAs that were found to be differentially active. The ten miRNAs that were found to be the most differentially active in MS PBMCs were all found to be less active in MS compared to healthy control. For five of them, activity and expression had a similar pattern, that is: these miRNAs are known to be down-regulated or under-expressed in MS compared to healthy control, or in severe MS compared to mild MS. Two of them are known to be over-expressed in certain stages of MS compared to healthy control, indicating a more complex working mechanism that may lead to disagreement between activity and expression. For the remaining three miRNAs, we could not find literature regarding their expression in MS.

While miRNA expression and activity are obviously related, they are not unanimous due to the complexity of cell biology. Our findings shed light on the interrelation of miRNA expression and activity. Through the integration of miRNA expression measurement and activity inference using our approach and code package, researchers can gain deeper insight into cellular level regulatory pathways, leading to a better understanding of spatial and cell-level biology.

In summary, miTEA-HiRes provides a means to capture the signal of miRNA activity, which usually goes undetected in gene expression datasets. Our study demonstrated that miTEA-HiRes successfully identified numerous functional miRNAs in specific samples and sample types. Through comparison with other methods, we emphasize the strengths of miTEA-HiRes. Most notably miTEA-HiRes provides robust quality of results while relying on the data itself without the need to collect and pre-train on large problem-specific datasets. miTEA-HiRes also offers ease of usage, with a friendly end-to-end pip-installable Python package. Additionally, we provide several leads that may be further investigated. miTEA-HiRes can thus help define miRNA roles in biological processes and point out miRNAs that should be further explored. Also, future work could utilize the general framework of our technique in uncovering the activity of transcription factors, providing a broader and more comprehensive understanding of active biological processes.

## Methods

### Statistical assessment of miRNA activity

The computation of activity *p*-values by miTEA-HiRes (including data preprocessing) is described as a pseudo-code in Supplementary Algorithm 1. miTEA-HiRes accepts as input both spatial transciptomics and single-cell RNA-sequencing raw count matrices containing gene counts per spot or cell. Bulk data may be analyzed as single cell data. The steps and components of the analysis are further described herein.

#### miRNA-target interactions (MTIs)

MTIs for humans and mice were acquired from the experimentally validated miRNA-target interactions database miRTarBase 8.0^117^. Non-functional MTIs were excluded, and functional MTIs with a strong experimental evidence or at least two weak experimental evidences were preserved, similar to the approach used in a previous study^63^. The filtered MTIs are provided within the miTEA-HiRes package for 706 mouse miRNAs and 2571 human miRNAs.

#### Preprocessing

For single-cell data, if there are multiple files, they are merged into a single matrix and a random sample of 10,000 cells is taken to improve performance and minimally sacrificing statistical power. After data cleansing (i.e. removing duplicated genes, removing genes with zero counts, and removing cells with identical gene expression profiles), miTEA-HiRes normalizes the count matrix so that the total number of reads in each cell or spot is 10,000 (Fig. 1, top middle panel).

To capture deviations in gene expression relative to its own dynamic expression across samples, each gene’s values are transformed into z-scores (Fig. 1, top right panel). That is, for a dataset with *S* cells or spots, each count *c*(*g*, *s*) per gene\transcript *g* and cell\spot *s* is transformed as follows:

$$z(g,s)=\frac{c(g,s)-a(g)}{\sigma (g)}$$

Where

$$a(g)=\frac{1}{S}{\sum}_{s}c(g,s)$$

and

$$\sigma (g)=\sqrt{\frac{1}{S}{\sum}_{s}{(c(g,s)-a(g))}^{2}}$$

Automatic data preprocessing is available in the miTEA-HiRes package.

#### Activity *p*-values

As depicted in Fig. 1 (middle row), for each spot or cell *s*, miTEA-HiRes ranks the genes\transcripts *g* by their z-score transformed counts, with the smallest value *z*(*g*, *s*) at the top and the largest value *z*(*g*, *s*) at the bottom. Next, using the set of MTIs, miTEA-HiRes converts this ranked gene list into a binary ordered list for each miRNA of interest, where target genes are assigned a value of 1 and all other genes are assigned a value of 0 (as depicted in Supplementary Fig. 3). miTEA-HiRes then employs the mHG (minimum hypergeometric) test (see next section) to assess statistical enrichment in this ranked binary list. For this purpose, we are using the XL-mHG Python package^118,119^. The mHG *p*-value is then used by miTEA-HiRes to represent the miRNA activity level for the specific combination of miRNA and cell or spot being analyzed. In case no targets are identified, the *p*-value generated by the mHG test is exactly one.

#### mHG (minimum hypergeometric) test

The nonparametric mHG test was developed by ref. ^65^ and is only briefly described here for completeness. The test aims to evaluate the level of enrichment present at the top of a ranked binary list. According to the null hypothesis, the given list is randomly and uniformly selected from a pool of all lists containing N entries, with K of them being 1s. The alternative hypothesis suggests that the 1s have a tendency to prominently appear at the top of the list.

Let

$$X \sim Hypergeometric(N, K, n)$$

and let *λ* be the observed binary list of length *N*, containing *K* 1s and (*N*−*K*) 0s. The mHG statistic is computed as:

$$mHG (\lambda) = {min}_{1 \le n \le N} Prob (X \ge b_n(\lambda))$$

Where

$${b}_{n} = {\sum}_{i=1}^{n}{\lambda }_{i}.$$

The *p*-value of mHG is then computed as the fraction of permutations $\tilde{\lambda }$, of the vector *λ*, for which $mHG(\tilde{\lambda })\le mHG(\lambda )$. That is, when using *Λ* to denote all such permuted patterns, we consider:

1 $$\,{{\mbox{p-value}}}\,\left(mHG(\lambda )\right)=\frac{\left| \left\{\tilde{\lambda }\in \Lambda | mHG(\tilde{\lambda })\le mHG(\lambda )\right\}\right| }{\left(\begin{array}{c}N \\ K\end{array}\right)}$$

#### Activity scores

According to the data type and mode of operation (spatial, or single cell: *total activity* or *comparative activity* modes), miTEA-HiRes ranks miRNAs to highlight the most notable ones, and assigns an aggregated activity score for each one of them (details follow). Relevant miRNA activity plots are produced, with activity *p*-values (per cell/spot) transformed to -log10(*p*-value) to emphasize significant results (see Fig. 1, bottom row). The ranked list of miRNAs, as well as links to the corresponding plots, are provided by the miTEA-HiRes package.

#### For spatial transcriptomics

The aggregated activity score for each miRNA is the fraction of spots with observed significant activity (*p*-value  < 1*e*−5). miRNAs are sorted based on their activity scores, and spatial miRNA activity maps are generated as part of the output.

#### For single-cell RNA-seq in *total activity* mode

miTEA-HiRes first averages the activity *p*-values across all cells for each miRNA. Then, these average *p*-values are FDR-corrected to obtain the final activity score for every miRNA. Also, miTEA-HiRes produces activity maps on a UMAP layout. In order to treat the activity score as an exact *p*-value, a more conservative merging approach may be applied^120–124^.

#### For single-cell RNA-seq in *comparative activity* mode

The miRNA activity score represents the level of differential activity between the two populations and is defined by the two-sided WRS *p*-value, subjected to FDR correction. We recommend using the *comparative activity* mode to compare populations of at least 100 cells. The ranking mechanism of miRNAs was designed to highlight miRNAs that are not only differentially active but also have consistently high activity *p*-values in at least one population. At the top of the ranked list are miRNAs that demonstrate an average activity *p*-value that is equal to or greater than the 0.97 quantile in at least one population and have activity scores smaller than 1*e*−8. Following these top miRNAs, are miRNAs that manifest an average activity *p*-value that is equal to or greater than the 0.9 quantile in at least one population and have activity score smaller than 1*e*−8. All the remaining miRNAs are sorted based on their activity scores.

Finally, relevant UMAPs and histograms are produced to demonstrate the different activity patterns across the two populations.

#### UMAP projection

Before computing the UMAP coordinates, miTEA-HiRes first performs commonly used preprocessing steps on the raw count matrix: cells with less than 200 genes are excluded, genes expressed in less than 3 cells are excluded, cells with total number of counts below the bottom 2% and above the top 2% are excluded to remove outliers, cells are normalized to 10,000 total counts, and all counts are transformed to log(count). miTEA-HiRes then follows the Scanpy recommended pipeline^125^ for generating UMAP, including the extraction of highly variable genes, PCA (principal component analysis), computation of the neighborhood graph of cells, and finally computation of UMAP coordinates.

### miTEA-HiRes Python package

miTEA-HiRes is a user friendly python package, available in https://github.com/EfiHerbst31/miTEA-HiResGithub and installable using pip. It can be executed with merely a path to the data and dataset name, but also has multiple options for more refined analysis. Basic execution includes end-to-end miRNA activity map computation. Output of the basic execution includes an activity matrix, an html list of ranked miRNAs, along with their activity scores and links to plots of top 10 miRNAs. Executions can be done via command-line or by importing the library and using its functions. Data type (i.e. single-cell or spatial transcriptomics), and data extension (supported: txt, tsv, pkl and mtx; files may be zipped) are automatically inferred from the data location. If preprocessing is required, multiple files are merged, 10,000 cells are sampled by default and data cleaning is performed (processed data is saved as .pkl file for reproducibility). Supported species are *homo sapiens* and *mus musculus*. In order to run in single-cell *comparative activity* mode, populations strings contained within cell names are required, which are assumed to be unique across the cells. miTEA-HiRes is computation intensive (for example, the running time of a spatial dataset with 2264 spots and  ~6000 genes, on a 16 cores machine with 706 examined microRNAs, is 22 minutes), hence it is recommended to run miTEA-HiRes on a multicore machine (by default all available CPUs are utilized). By default all miRNAs available in the MTI database are analyzed. By default top 10 most interesting miRNAs are plotted, however this can be changed. More fine-tuning can be done using the possibility to filter spots with a small number of reads, and with the possibility to change the threshold below which a spot is considered to be active. Debugging mode allows for detailed output. Detailed information is provided in the package https://github.com/EfiHerbst31/miTEA-HiResreadme document. Packages utilized within miTEA-HiRes: Anndata^126^, Scanpy^127^ and XL-mHG^119^.

### Statistics and reproducibility

#### Analyzing spatial transcriptomics data sets

Spatial transcriptomics datasets were analyzed using miTEA-HiRes without any preprocessing, using the default parameters.

#### Analyzing Multiple Sclerosis single-cell data sets

Both PBMC and CSF datasets were analyzed using miTEA-HiRes without any preprocessing, using default parameters (sampling 10K cells). The MS dataset with cell types was analyzed without sampling, to improve statistical significance when analyzing cell subsets.

#### Analyzing breast cancer single-cell data sets

To enhance the statistical power and leverage the similarities between breast cancer samples, we merged all cell lines and patient-derived cells into two groups: 1182 static cells and 1992 migratory cells. We then used the miTEA-HiRes analysis pipeline.

#### GDC data curation

First, we used the GDC portal^106^ to create two cohorts- one of cases with RNAseq data and the other of cases with miRNAseq data. We then used Python code to intersect these two cohorts, and loaded the intersected cohort back to the GDC portal. We used the portal to identify the RNAseq samples and the miRNAseq samples that followed these criteria: primary tumor, solid tissue, open access, tsv or txt. We then used Python to download the relevant samples. If more than one RNAseq sample \miRNAseq sample was available for the same case, we chose the one with the most detected genes \miRNAs. Finally, we produced one gene expression count matrix of 11,927 samples and 57,287 genes from the RNAseq samples, and one miRNA expression (reads per million) count matrix of 11,927 samples and 1810 miRNAs from the miRNAseq samples. We then found that in the second matrix, for some miRNAs, we had the expression values of the precursors of the miRNA (marked with “-1","-2"... at the end) rather than the mature miRNA. In these cases, we summed the expression values of the precursors to get an assessment of the expression of the mature miRNA. After performing this step, and removing miRNAs that were not expressed at all, we had 1662 miRNAs in the miRNA expression matrix.

#### Evaluating agreement between activity and expression in the GDC dataset, for miTEA-HiRes and miReact

We ran miTEA-HiRes on the gene expression count matrix using the *total activity* mode (Methods). We also ran miReact^59^ in R on the same gene expression count matrix, as described in miReact tutorial^128^. From both methods we received an activity *p*-values matrix as output, with a prediction of the activity of each miRNA (2571 miRNAs for miTEA-HiRes, 2578 miRNAs for miReact) in each of the 11,927 samples. We transformed the miTEA-HiRes activity *p*-values matrix to -log10(*p*-values) (the miReact activity *p*-values were already -log10 transformed). Each of the miRNAs that appeared in the activity matrices of both miTEA-HiRes and miReact, also appeared as one of the 1662 miRNAs in the miRNA expression matrix. For strand-specific miRNAs, no strand was specified for the miRNA that appeared in the miRNA expression matrix.

For both miTEA-HiRes and miReact, we determined activity as follows. A miRNA was considered active in a certain sample if its activity *p*-value was less than 0.05. If a strand-specific miRNA was considered active, we considered the no-strand version of the same miRNA (the one that appeared in the miRNA expression matrix) as active. For each sample, we ordered the miRNAs based on their descending expression. We then used the mHG test to evaluate whether active miRNAs were enriched at the top of the list. The *p*-values of the mHG tests performed for all samples are summarized in Fig. 5.

Similarly, we considered miRNAs with an activity *p*-value of more than 0.1 as non-active. If a strand-specific miRNA was found to be non-active, and its opposite strand was not found to be active, then we considered the no-strand version of the same miRNA (the one that appeared in the miRNA expression matrix) to be non-active. For each sample, we ordered the miRNAs based on their ascending expression. We then used the mHG test to evaluate whether non-active miRNAs were enriched at the top of the list. The *p*-values of the mHG tests performed for all samples are summarized in Fig. 5.

#### Activity and expression across cancer tissues of origin

We downloaded the clinical data for the final set of cases, chosen from the GDC portal as explained above. Five cases had multiple entries (with inconsistencies in the relevant column) and were excluded. On the “tissue_or_organ_of_origin" column, we found that 11,896 cases had 140 tissues of origin, while for 26 cases the tissue of origin was not reported. We used a Python function to group the origins into 35 combined tissues. For example, “Fallopian tube", “Cervix uteri" and “Endometrium" were classified as “Female Reproductive System", while “Cortex of adrenal gland" and “Medulla of adrenal gland" were classified as “Adrenal Gland". Then, for miR-101-3p, miR-22-3p, and miR-10b-3p, we produced the box plots of miTEA-HiRes activity values across 14 combined tissues that had at least 200 samples each (Fig. 5). Similarly, we produced the expression box plots for the same miRNAs (Supplementary Fig. 6).

#### Evaluating the relationship between single-cell miRNA expression and activity

We downloaded total-RNA sequencing datasets obtained using the Smart-seq-total method^45^. We then merged the datasets of human primary fibroblasts, HEK293T, and MCF7 cells into a single human dataset (633 cells), and the datasets of all stages of murine embryonic stem cells differentiation into a single mouse dataset (2167 cells). To explore the enrichment level of active miRNAs at the top of the ranked list of overexpressed miRNAs, we performed the following processing pipeline. To obtain the list of most expressed miRNAs, we summed the raw counts for each miRNA over all the cells. We retained only those miRNAs that were also available in miTEA-HiRes database. We ranked this list in descending order, with the most expressed miRNAs positioned at the top. A miRNA was considered active if its activity score was less than 0.05 (average activity *p*-value over all cells). miTEA-HiRes’ MTI database distinguishes between miRNAs with 3p and 5p strands, while the total-RNA dataset displays only one value for the two strands, which represents the summed expression of them. To account for this, we considered a miRNA to be active if at least one of the strands was active. To utilize the mHG test, we converted the ranked list of overexpressed miRNAs into 1’s (representing active miRNAs) and 0’s.

To explore the enrichment level of non-active miRNAs at the top of the ranked list of underexpressed miRNAs, we performed the following processing pipeline. To obtain the list of underexpressed miRNAs, we used the reverse-order of the overexpressed miRNAs list obtained previously. A miRNA was considered to be non-active if its activity score was greater than 0.1 (average activity *p*-value over all cells). If a miRNA was found to be non-active, but its other strand (3p or 5p) was previously considered as active, it was excluded from the non-active miRNAs list to ensure that only truly non-active miRNAs were included. To utilize the mHG test, we converted the ranked list of underexpressed miRNAs into 1’s (representing non-active miRNA) and 0’s otherwise.

For the human dataset, enrichment of active miRNAs at the top of the list of expressed miRNAs was calculated as follows: *p*_*mHG*_(*N* = 1462, *B* = 740, n* = 138, *b*(n*) = 97) = 3.76*e*−05. Enrichment of non-active miRNAs at the bottom of the list: *p*_*mHG*_(*N* = 1462, *B* = 526, n* = 1367, *b*(n*) = 508) = 0.0055. For the mouse dataset, enrichment of active miRNAs at the top of the list: *p*_*mHG*_(*N* = 492, *B* = 47, n* = 234, *b*(n*) = 32) = 0.0301. Enrichment of non-active miRNAs at the bottom of the list: *p*_*mHG*_(*N* = 492, *B* = 413, n* = 265, *b*(n*) = 236) = 0.0114). N refers to the number of shared miRNAs between the dataset and miTEA-HiRes database, B is the number of active (non-active) miRNAs, n* is the cutoff selected by the mHG test, b(n*) is the number of miRNAs both highly expressed and active (lowly expressed and non-active) at the cutoff.

#### Evaluating differential activity in single-cell miRNA induction experiment

We obtained the count matrices of 3280 distinct i199 cells, and 3143 i199-KTN1 cells, including induced and uninduced annotations directly from the author^114^. Following miRSCAPE methods^60^, we annotated the cells according to GeGFP-00001 expression levels (’induced’ if the expression level was within the top 20%, and ’uninduced’ if the expression level was zero). After cell annotation, we removed the genes related to miRNA induction experiment from the data. As a preprocessing step, we filtered out cells having gene detection rate amongst the top 2%. Also, as we noted that the overall gene detection rate was lower in the i199-KTN1 cells compared to the i199 cells, we filtered out cells having less than 5000 detected genes. As a result, we were left with the following cell type composition: i199 had 695 induced cells, 1561 uninduced cells, and 2814 cells in total. i199-KTN1 had 611 induced cells, 1075 uninduced cells, and 2264 cells in total. We then applied miTEA-HiRes in *comparative activity* mode, focusing on miR-199a-5p and miR-199a-3p. miTEA-HiRes indicated correctly the excess activity of both miRNAs in the induced cell group in both cell types, as measured using WRS *p*-values. Results are available at Zenodo 10.5281/zenodo.10720979.

### Reporting summary

Further information on research design is available in the Nature Portfolio Reporting Summary linked to this article.

## Supplementary information

Transparent Peer Review file

Supplementary Information

Reporting Summary

## Data Availability

Spatial transcriptomics read counts and spatial coordinates were downloaded from the Visium website^74^ for the following tissues: mouse brain^68^, human breast cancer^69^, human skin melanoma^70^, human lung cancer^71^, human ovarian cancer^72^ and human cerebellum^73^. The Multiple Sclerosis (MS) raw count matrices were downloaded from GEO repository under the accession number https://www.ncbi.nlm.nih.gov/geo/query/acc.cgi?acc=GSE138266GSE138266^77,129^. The MS processed count matrices, with cell type annotations, were downloaded from https://github.com/chenlingantelope/MSscRNAseq2019.git^130^. The breast cancer migratory and stationary count matrices were downloaded from GEO repository under the accession number https://www.ncbi.nlm.nih.gov/geo/query/acc.cgi?acc=GSE162726GSE162726^86,131^. The totalRNA human and mouse datasets were downloaded from the GEO repository under the accession number https://www.ncbi.nlm.nih.gov/geo/query/acc.cgi?acc=GSE151334GSE151334^45,132^. The count matrices of the miRNA induction experiments that were used for validation, were obtained directly from the authors^114^. Bulk cancer data was downloaded from the GDC portal https://gdc.cancer.gov/^106^ (see Methods for detailed instructions).

## Code availability

miTEA-HiRes’s code and instructions are available at: https://github.com/EfiHerbst31/miTEA-HiRes. Pip install is available at: https://pypi.org/project/mitea-hires/Full miTEA-HiRes results for the datasets in this paper, as well as a table of miRNA target lists pair-wise overlap (calculated by Jaccard index), can be found at Zenodo 10.5281/zenodo.10720979^133^.
