# Supplementary material for: Inferring single-cell and spatial microRNA activity from transcriptomics data
Source: Commun Biol. 2025 Jan 18;8:87. doi: 10.1038/s42003-025-07454-9 (PMC11743151; doi:10.1038/s42003-025-07454-9)
Supplement: Supplementary file 2 — Supplementary Information [file 42003_2025_7454_MOESM2_ESM.pdf]

Supplementary information

Statistical characteristics of miRNA-target interaction sets

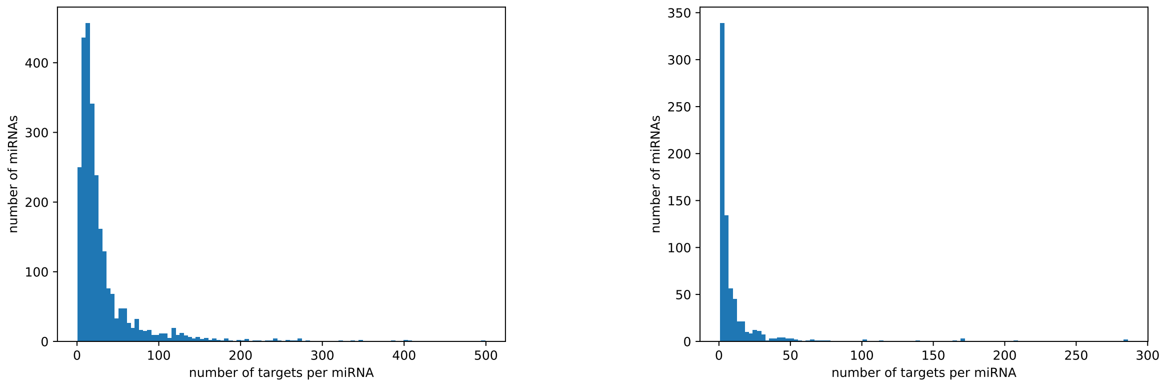

(a)

(b)

Supplementary Fig. 1: Histograms of number of targets per miRNA in the filtered human (**a**) and mouse (**b**) lists of miRNA-targets interactions. Number of miRNAs: human: 2571, mouse: 706. Median number of targets per miRNA: human: 17.0, mouse: 4.0. Average number of targets per miRNA: human: 30.3, mouse: 10.6.

Pseudo-code: computation of activity p-values

Supplementary Algorithm 1 miTEA-HiRes: Computation of activity p-values

input: C, M, $\{I(m)\}_{m\in M}$, $mHG()$

C is a count matrix, consisting of the count values $\{c(g,s)\}_{g\in G,s\in S}$.

G is the set of genes (rows) and S is the set of samples (columns).

M is a list of miRNAs.

$I(m) \subseteq G$ is a list of known targets of the miRNA m.

$mHG()$ is a function that performs the minimum-HyperGeometric test, that is: it accepts a vector of 1's and 0's and returns an mHG p-value, which attests to the level of over-enrichment of 1's at the top of the vector.

output: An activity matrix P with miRNAs as rows and samples as columns, consisting of activity p-values $\{p(m,s)\}_{m\in M,s\in S}$.

$n(g,s) = 10000 \cdot \frac{c(g,s)}{\sum_g c(g,s)} \quad \forall g,s$ // sample normalization

$z(g,s) = \frac{n(g,s) - \frac{1}{S}\cdot\sum_s n(g,s)}{\sqrt{\frac{1}{S}\sum_s \left(n(g,s) - \frac{1}{S}\cdot\sum_s n(g,s)\right)^2}} \quad \forall g,s$ // gene z-score transformation

for $m \in M$ **do**

    for $s \in S$ **do**

        $\forall i \in \{1,\dots,|G|\}$: $r(i) = g$ such that $z(g,s) > z(\tilde{g},s) \forall\ \tilde{g} \in \{r(j)\}_{j=1}^{i-1}$ // sort the list of genes by an ascending order of $\{z(g,s)\}_{g\in G}$

        $\forall i \in \{1,\dots,|G|\}$: $x(i) = \begin{cases} 1 & \text{if } r(i) \in I(m) \\ 0 & \text{else} \end{cases}$ // create a binary vector

        $p(m,s) = mHG(x)$ // compute mHG p-value

    end for

end for

return $\{p(m,s)\}_{m\in M,s\in S}$

ANLN gene count distribution

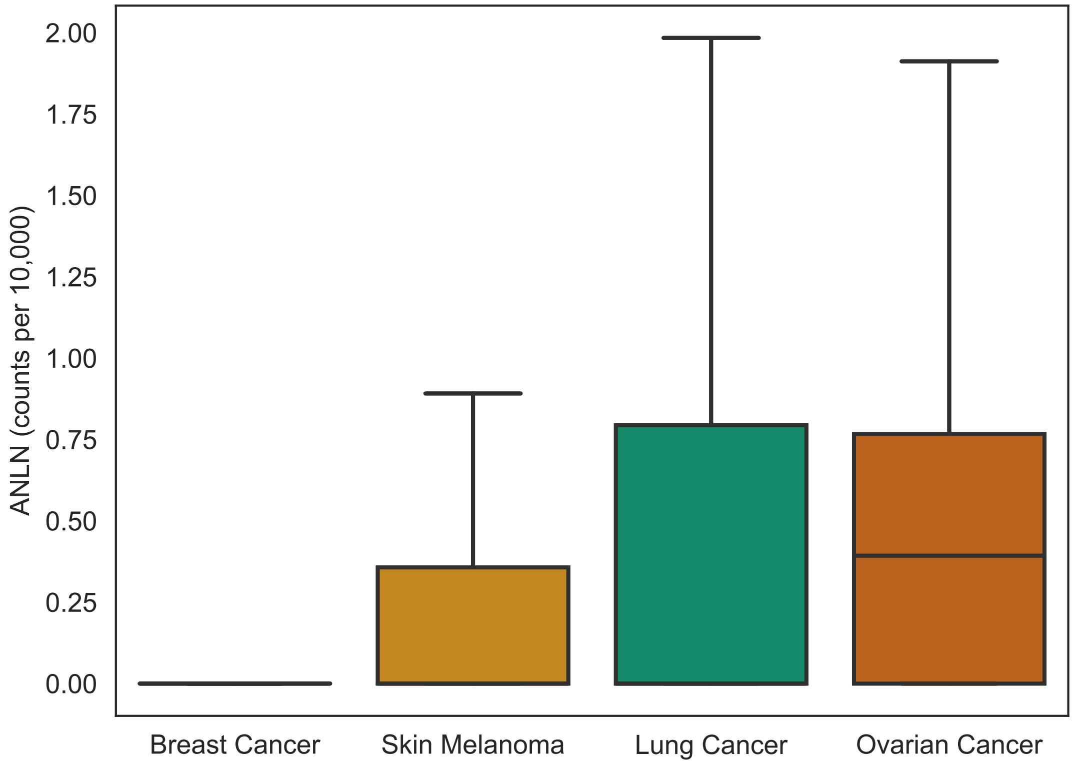

Supplementary Fig. 2: *ANLN* count distribution across several human cancer conditions. Outliers are not shown in the plot. Human and mouse brain tissues were excluded for not containing *ANLN* reads. Spatial transcriptomics data was obtained from the Visium website[1–4].

Examples for target enrichment in real cells

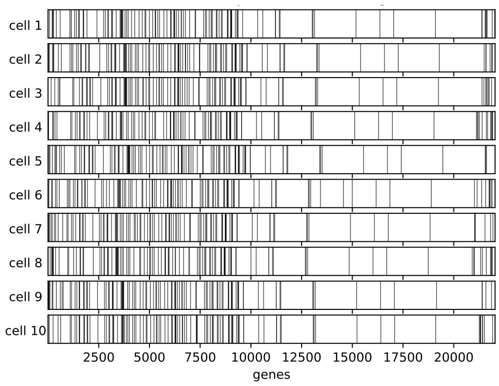

(a)

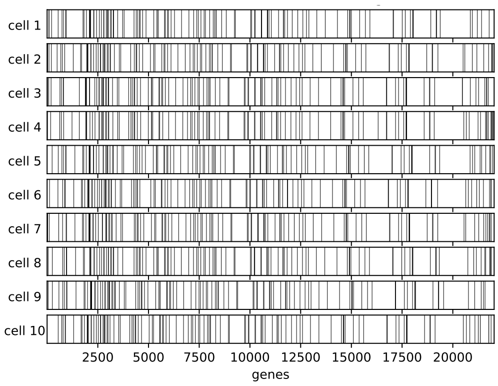

(b)

Supplementary Fig. 3: Target enrichment in 10 random cells from the MS PBMCs dataset[5] for two miRNAs. In every cell genes are sorted post normalization and z-score transformation. Black lines stand for target genes. An active miRNA and an inactive miRNA (as reported by miTEA-HiRes) are depicted in panels **a** and **b** respectively. **a** miR-372-3p with 112 targets. *total activity* mode: activity score: $8.25e-08$; rank: 15. **b** miR-3924 with 117 targets. *total activity* mode: activity score: $6.11e-02$; rank: 725.

miTEA-HiRes analysis of a single-cell dataset of CSF cells obtained from patients with Multiple Sclerosis and a control group

miRNA	*total activity* mode		*comparative activity* mode			Literature
	rank	activity score	rank	activity score	Histogram of activity p-values	
miR-93-5p	1	2.03e-08	1	6.06e-156	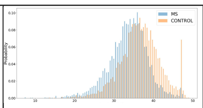	Regulates MS risk genes[6]; Significant over-expression of miR-93-5p in PBMCs of RRMS patients compared to healthy subjects[7]; belongs to the miR-106b/25 cluster[8]
miR-106b-5p	2	5.04e-08	2	4.75e-123	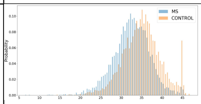	Increased MS related disability is associated with down-regulation of miR-106b-5p and miR-19b-3p in RRMS patients[9]; belongs to the miR-106b/25 cluster[8]
miR-16-5p	3	4.06e-07	4	4.47e-37	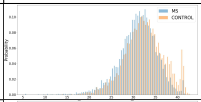	Underexpressed in PBMCs, CD4+ T cells and B cells of RRMS patients, comparing to healthy subjects[10]
miR-19b-3p	4	3.75e-07	8	4.16e-75	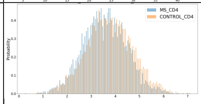	Increased MS related disability is associated with downregulation of miR-106b-5p and miR-19b-3p in relapsing remitting MS patients[9]; belongs to the miR-106a/363 cluster[11]
miR-19a-3p	5	3.42e-07	>10	-	-	Belongs to the miR-17/92 cluster[12]
miR-92a-3p	6	3.20e-07	>10	-	-	Belongs to the miR- 17/92 cluster[12]
miR-155-5p	7	2.75e-07	10	1.64e-15	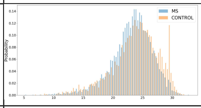	miR-155 is a crucial regulator of inflammation, modulates the autoimmune response in MS and affects the function of the brain-blood barrier in MS patients[13]
miR-32-5p	8	8.36e-07	>10	-	-	Associated with MS severity[14]
miR-17-5p	9	1.14e-06	5	1.37e-122	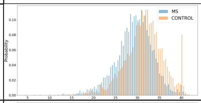	Inhibits T Cell activation genes, and underexpressed in MS whole blood[15]; belongs to the miR-17/92 cluster[12]
miR-106a-5p	10	3.78e-06	9	4.30e-88	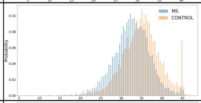	Belongs to the miR-106a/363 cluster[11]
miR-20a-5p	>10	-	3	2.36e-151	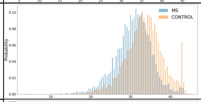	inhibits T Cell activation genes, and underexpressed in MS whole blood[15]; belongs to the miR-17/92 cluster[12]
miR-519d-3p	>10	-	6	1.91e-123	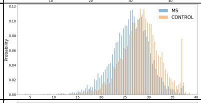	No literature found
miR-20b-5p	>10	-	7	1.91e-117	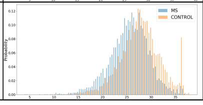	Belongs to the miR-106a/363 cluster[11]

Supplementary Table 1: miTEA-HiRes analysis of MS single cell dataset of CSF (4,998 and 4,164 cells from MS and control groups, respectively). Top 10 most overall active miRNAs as computed by miTEA-HiRes in *total activity* mode, and top 10 differentially active miRNAs as computed by miTEA-HiRes in *comparative activity* mode: MS vs. control. In the histograms the x-axes indicate -log10(activity p-values). Histogram legend: Blue: MS, orange: control. In *comparative activity* mode, miRNAs are ranked as described in Methods, Activity scores section.

Additional miTEA-HiRes analysis results: single-cell dataset of cell-type specific PBMCs and CSF obtained from patients with Multiple Sclerosis and a control group

Cell type and origin	miR-19a-3p	miR-519c-3p	miR-519b-3p	miR-133a-3p	miR-6504-3p	miR-582-5p	miR-2681-5p	miR-329-3p	miR-124-3p	miR-5590-3p
CD8n PBMCs	1.55e-09 ↓	1.77e-18 ↓	8.34e-17 ↓	1.47e-18 ↓	1.50e-17 ↓	1.29e-22 ↓	1.58e-18 ↓	1.90e-07 ↓	2.53e-26 ↓	4.45e-17 ↓
CD8a PBMCs	2.56e-41 ↓	7.48e-22 ↓	4.55e-21 ↓	7.28e-48 ↓	4.36e-51 ↓	5.95e-21 ↓	7.89e-58 ↓	5.84e-23 ↓	1.15e-126 ↓	9.10e-63 ↓
CD4 PBMCs	6.80e-42 ↓	7.88e-57 ↓	9.60e-54 ↓	7.76e-56 ↓	5.15e-57 ↓	1.63e-96 ↓	6.38e-95 ↓	6.83e-22 ↓	3.12e-97 ↓	7.18e-46 ↓
NK1 PBMCs	7.96e-16 ↓	1.42e-16 ↓	7.46e-16 ↓	3.68e-05 ↓	7.19e-20 ↓	1.32e-10 ↓	1.60e-25 ↓	4.90e-05 ↓	5.63e-41 ↓	3.63e-14 ↓
B1 PBMCs	4.20e-09 ↓	5.93e-14 ↓	6.92e-14 ↓	1.88e-04 ↓	1.24e-09 ↓	1.77e-10 ↓	1.56e-08 ↓	2.41e-10 ↓	7.26e-23 ↓	9.57e-02
B2 PBMCs	5.93e-07 ↓	1.68e-09 ↓	2.85e-09 ↓	1.35e-05 ↓	2.47e-07 ↓	3.06e-05 ↓	2.30e-06 ↓	4.40e-06 ↓	8.78e-06 ↓	7.76e-02
Tregs PBMCs	1.17e-03 ↓	2.00e-02	1.39e-02	6.03e-07 ↓	3.72e-08 ↓	1.43e-07 ↓	3.47e-14 ↓	5.33e-08 ↓	6.54e-22 ↓	6.39e-06 ↓
Gran PBMCs	3.28e-06 ↓	7.55e-01	2.00e-01	1.27e-01	6.97e-03 ↓	2.06e-07 ↓	3.50e-05 ↓	5.70e-01	8.84e-03 ↑	4.73e-03 ↓
Tdg PBMCs	5.92e-04 ↓	7.20e-05 ↓	3.33e-04 ↓	3.23e-03 ↓	1.71e-01	6.85e-01	6.71e-04 ↓	2.32e-01	2.57e-11 ↓	1.77e-03 ↓
Mono PBMCs	7.37e-08 ↓	8.30e-09 ↓	1.91e-07 ↓	4.92e-01	1.03e-09 ↓	1.78e-10 ↓	1.17e-03 ↓	2.65e-05 ↓	2.83e-01	8.62e-02
CD8a CSF	7.39e-16 ↓	7.13e-09 ↓	5.45e-09 ↓	5.99e-18 ↓	9.42e-30 ↓	4.78e-05 ↓	9.24e-09 ↓	7.16e-01	1.84e-26 ↓	1.45e-11 ↓
CD4 CSF	7.92e-22 ↓	9.93e-05 ↓	6.79e-04 ↓	1.24e-26 ↓	2.51e-80 ↓	2.23e-14 ↓	2.23e-04 ↓	8.94e-02	3.40e-02	1.79e-17 ↓
Tregs CSF	4.42e-02	4.80e-01	5.85e-01	3.42e-08 ↓	1.96e-07 ↓	2.65e-02	9.47e-01	2.53e-01	3.33e-01	5.03e-01

Supplementary Table 2: Comparison between MS and control in cell-type specific groups in PBMCs and CSF datasets. For each cell type, MS and control populations were analyzed by miTEA-HiRes in *comparative activity* mode. Cell-type keys: CD8n (non-activated CD8+ T cells), CD8a (activated CD8+ T cells), CD4 (CD4+ T cells), NK1 (natural killer cells), B1/B2 (B cell subsets), Tregs (regulatory CD4+ T cells), Gran (Granulocytes), Tdg ($\gamma\delta$ T cells), Mono (monocyte cells)[5]. ↓, ↑ represent reduced\elevated activity in MS compared to control, respectively. Top 10 results for all comparisons can be found at Zenodo. Group sizes can be found in Supplementary Table 4.

Cell types and origin	miR-4796-3p	miR-519c-3p	miR-519b-3p	miR-651-3p	miR-30a-3p	miR-329-3p
**CD4 Vs. B1**						
PBMCs control	4.20e-01	2.60e-01	9.84e-01	1.77e-05 ↓	6.25e-06 ↑	4.73e-03 ↓
PBMCs MS	4.15e-14 ↑	2.06e-02	2.51e-01	9.04e-03 ↑	7.52e-56 ↑	2.54e-11 ↓
**CD8a Vs. B1**						
PBMCs control	7.07e-02	1.00e-01	6.68e-01	1.72e-11 ↓	6.10e-01	2.75e-01
PBMCs MS	1.08e-46 ↑	7.48e-03 ↓	1.59e-01	2.63e-01	4.30e-04 ↑	2.42e-02
**CD4 Vs. B2**						
PBMCs control	1.90e-08 ↓	6.66e-02	1.22e-01	2.85e-06 ↓	1.71e-05 ↑	4.74e-03 ↓
PBMCs MS	8.75e-09 ↓	5.78e-13 ↓	7.50e-12 ↓	2.14e-06 ↓	1.52e-44 ↑	1.70e-15 ↓
**CD8a Vs. B2**						
PBMCs control	1.16e-04 ↓	2.67e-02	6.57e-02	1.19e-10 ↓	3.95e-02	5.82e-01
PBMCs MS	8.86e-01	1.10e-12 ↓	3.21e-11 ↓	1.64e-11 ↓	5.64e-12 ↑	1.58e-02
**CD8a Vs. Tdg**						
PBMCs control	3.60e-05 ↓	4.06e-02	2.88e-02	3.74e-04 ↓	1.75e-04 ↓	3.74e-02
PBMCs MS	6.77e-01	5.06e-02	8.17e-02	5.05e-02	3.32e-06 ↓	9.19e-10 ↑
**CD8a Vs. Tregs**						
PBMCs control	4.53e-02	2.41e-06 ↓	2.69e-05 ↓	2.38e-11 ↓	7.73e-06 ↓	3.55e-06 ↓
PBMCs MS	8.04e-01	9.70e-03 ↓	2.08e-02	1.22e-20↓	2.16e-19 ↓	1.22e-11 ↓
CSF control	9.75e-01	3.11e-05 ↓	3.66e-05 ↓	3.37e-02	3.58e-07 ↓	5.11e-01
CSF MS	5.96e-01	2.92e-01	4.42e-01	2.42e-04 ↓	5.10e-05 ↓	1.31e-02
**CD4 Vs. CD8a**						
PBMCs control	7.16e-05 ↓	3.69e-01	4.82e-01	4.89e-05 ↑	1.16e-07 ↑	2.16e-08 ↓
PBMCs MS	1.66e-28 ↓	3.75e-01	5.84e-01	2.10e-06 ↑	6.07e-41 ↑	6.21e-28 ↓
CSF control	8.77e-05 ↓	1.23e-01	6.36e-02	1.76e-01	3.03e-09 ↑	3.65e-24 ↓
CSF MS	2.37e-04 ↓	1.87e-02	1.29e-02	6.84e-06 ↑	1.20e-04 ↑	4.27e-17 ↓

Supplementary Table 3: Additional comparisons between various types of cells in the MS and control PBMCs and CSF datasets. Cell-type keys: CD8a (activated CD8+ T cells), CD4 (CD4+ T cells), B1/B2 (B cell subsets), Tregs (regulatory CD4+ T cells), Tdg ($\gamma\delta$ T cells)[5]. ↓, ↑ represent activity in the second population compared to the first one. For example, in the uppermost box, ↓ represents reduced activity in B1 cells compared to CD4+ T cells. Top 10 results for all comparisons can be found at Zenodo. Group sizes can be found in Supplementary Table 4.

Dataset	Group	Cell type	Number of cells	Percentage of cells
PBMCs	MS	all	25,831	100
		B1	1,729	6.7
		B2	656	2.5
		Gran	3,600	13.9
		Mono	849	3.3
		Tdg	569	2.2
		Tregs	983	3.8
		CD4	8,596	33.3
		CD8a	2,938	11.4
		CD8n	1,537	6
		NK1	2,619	10.1
	Control	all	17,138	100
		B1	921	5.4
		B2	316	1.8
		Gran	2,378	13.9
		Mono	447	2.6
		Tdg	424	2.5
		Tregs	504	2.9
		CD4	5,337	31.1
		CD8a	2,050	12
		CD8n	1,181	6.9
		NK1	1,020	6
CSF	MS	all	9,652	100
		B1	37	0.4
		B2	195	2
		Gran	94	1
		Mono	138	1.4
		Tdg	69	0.7
		Tregs	1,145	11.9
		CD4	5,096	52.8
		CD8a	1,741	18
		CD8n	117	1.2
		NK1	83	0.9
	Control	all	12,705	100
		B1	6	0
		B2	24	0.2
		Gran	241	1.9
		Mono	527	4.1
		Tdg	166	1.3
		Tregs	657	5.2
		CD4	6,076	47.8
		CD8a	3,321	26.1
		CD8n	89	0.7
		NK1	21	0.2

Supplementary Table 4: Breakdown of Multiple Sclerosis datasets into cell type specific groups. Cell-type keys: B1/B2 (B cell subsets), Gran (granulocytes), Mono (monocytes), Tdg ($\gamma\delta$ T cells), Tregs (regulatory CD4+ T cells), CD4 (CD4+ T cells), CD8a (activated CD8+ T cells), CD8n (non-activated CD8+ T cells), NK1 (natural killer cells)[5]. Annotated datasets were obtained from https://github.com/chenlingantelope/MSscRNAseq2019.git.

Additional miTEA-HiRes analysis results: single-cell dataset of migratory and stationary breast cancer cells

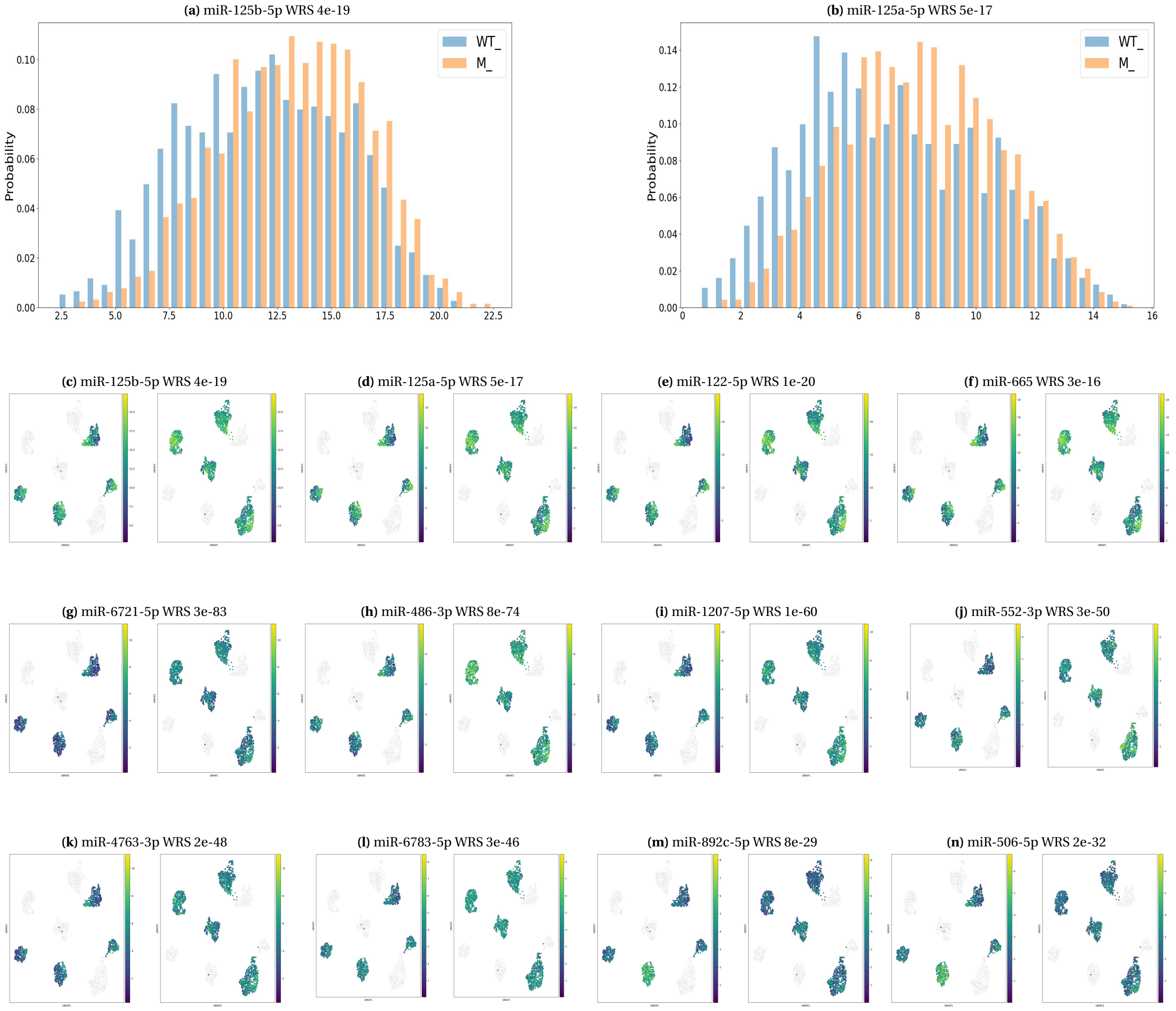

Supplementary Fig. 4: miRNA activity layouts for migratory and static breast cancer cells. **a,b** Histograms of activity values divided by populations, produced by miTEA-HiRes in *comparative activity* mode. In the histograms the x-axes indicate -log10(activity p-values). "WT_" refers to static cells, "M_" refers to migratory cells. **c-n** Comparative activity maps in a UMAP layout, produced by miTEA-HiRes in *comparative activity* mode. Left: static population, right: migratory population. Colors depict -log10(activity p-values). Full results are available at `https://doi.org/10.5281/zenodo.10720979`.

Enriched pathways for targets of miR-16-5p, miR-17-5p, miR-8485 and miR-124-3p

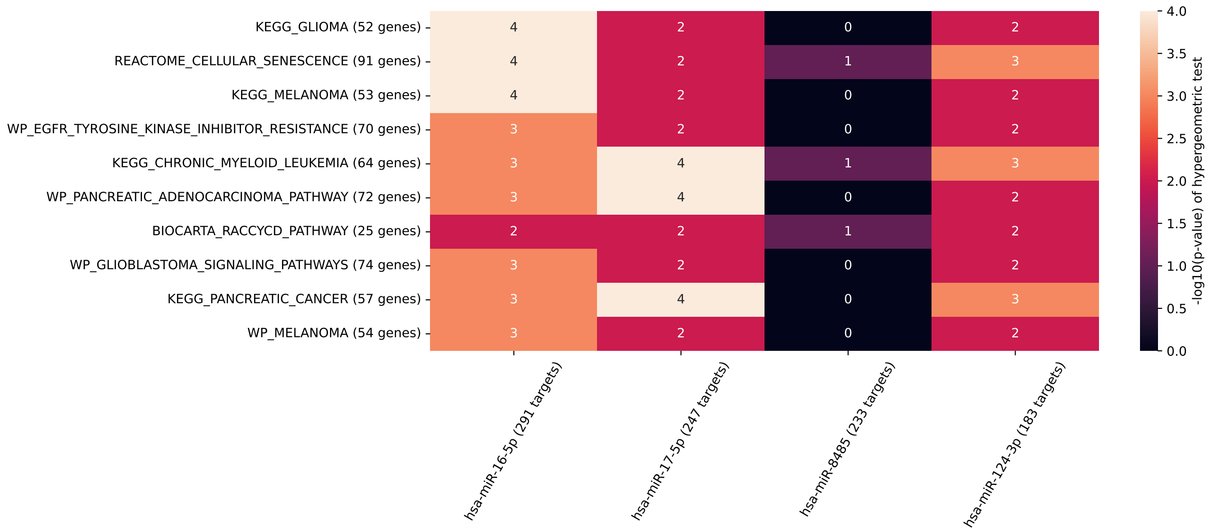

Supplementary Fig. 5: Gene set enrichment of canonical pathways among lowly expressed targets of four miRNAs that have similar expression patterns in the breast cancer metastasis dataset. Target lists were filtered to include only targets that had a median rank of 7000 or less (after data normalization and z-scoring; The dataset includes 29922 genes overall). Pathway gene sets were reduced to genes that were included in the dataset. Then, pathway gene sets with less than 10 genes were removed. Enrichment was calculated using the hypergeometric test, targets of all miRNAs were used as the background set. Pathway gene sets that were found to be enriched (hypergeometric p-value ≤ 0.01) in the target lists of at least three (out of four) miRNAs are presented.

Expression of example miRNAs in the GDC dataset, grouped by cancer tissue

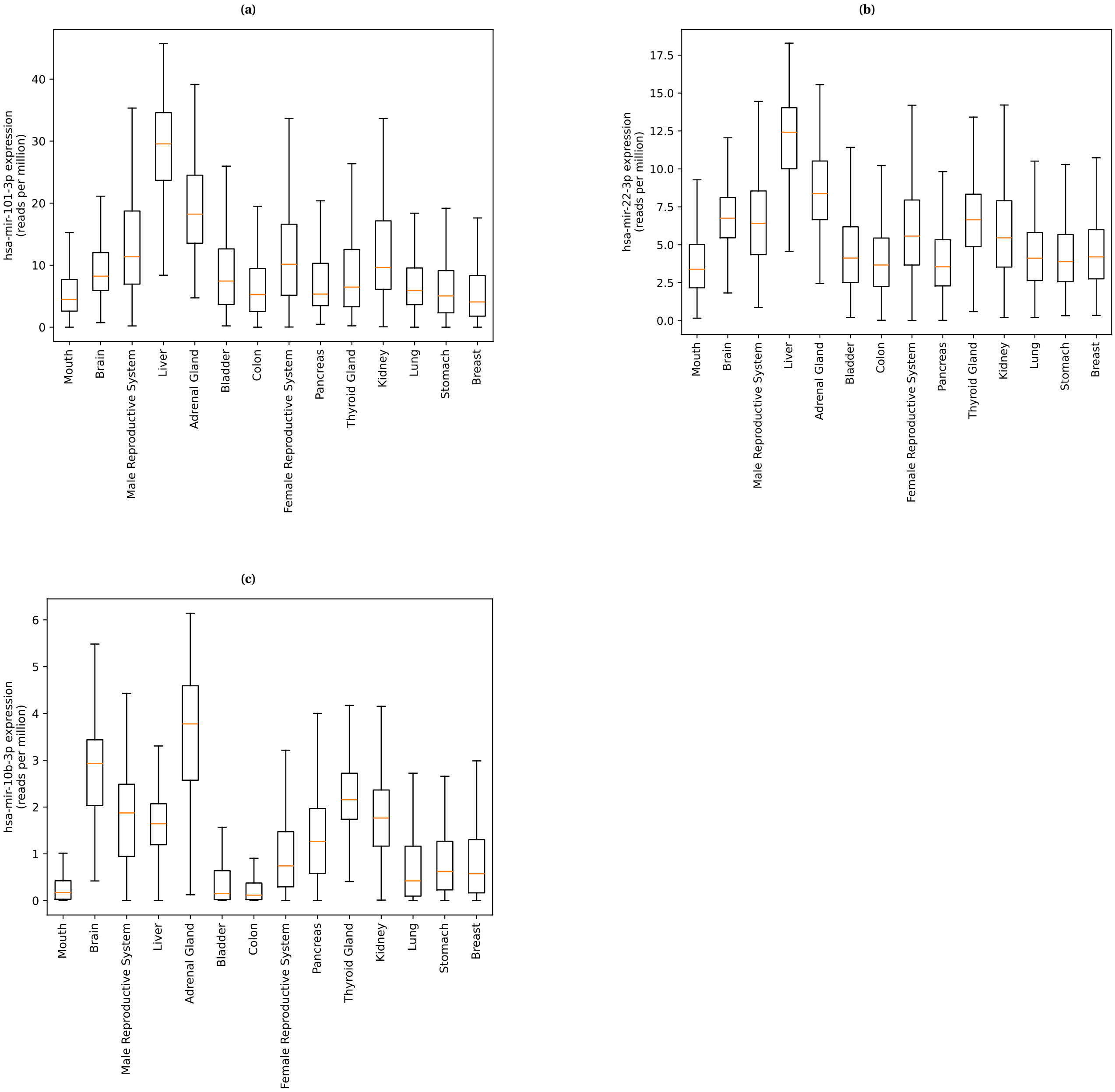

Supplementary Fig. 6: Expression of example miRNAs in the GDC dataset, grouped by cancer tissue. **a** miR-101-3p; **b** miR-22-3p; **c** miR-10b-3p.

Connection between expression and activity as appears in single-cell totalRNA datasets of human and mouse

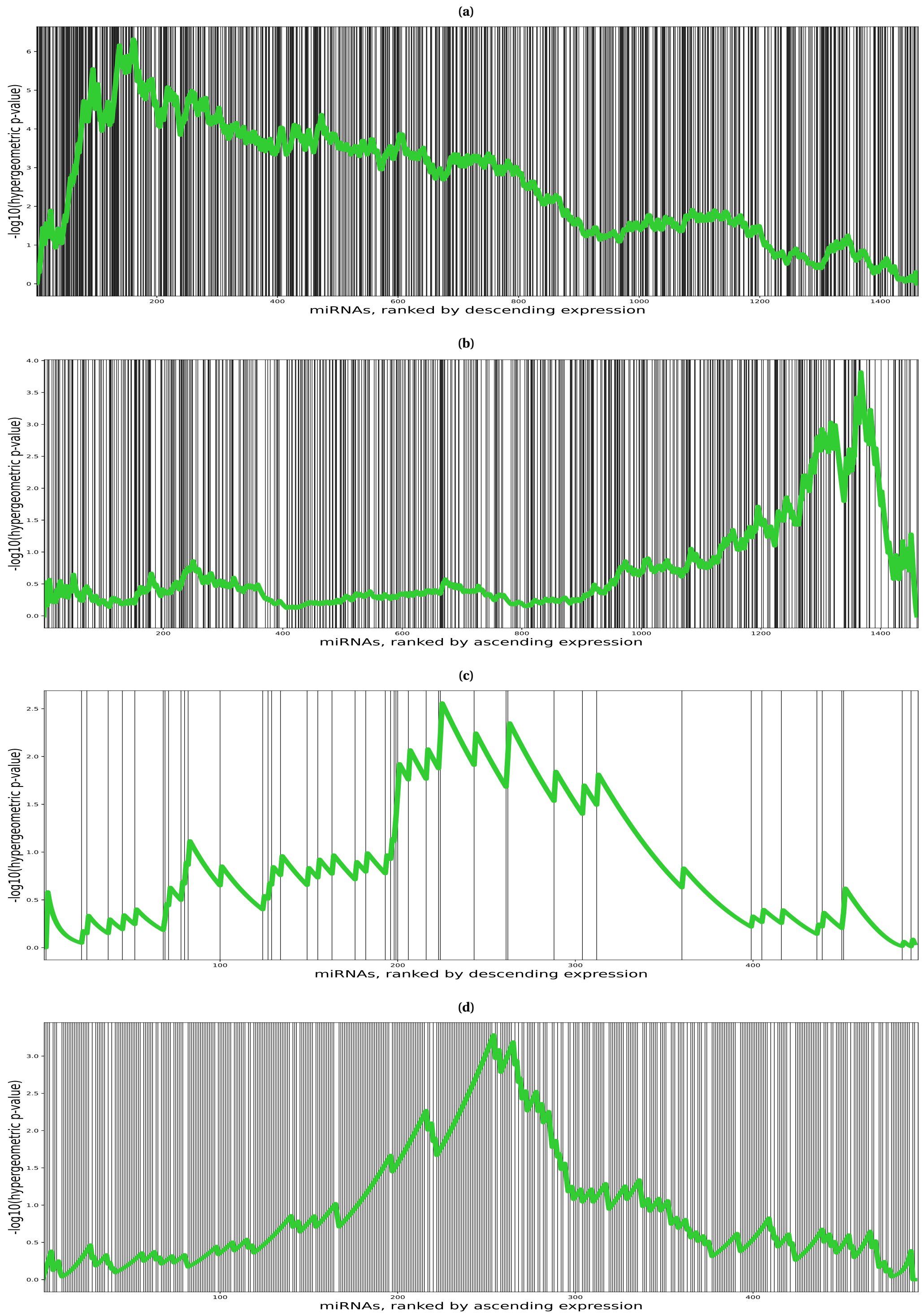

Supplementary Fig. 7: Enrichment plots, showing the relationship between expression and activity of miRNAs in totalRNA datasets. The green plot represents the -log10(p-value) of the hypergeometric test for the overlap between expressed and active miRNAs (or: not expressed and non-active miRNAs) at every point. For example: the green value at x=10 is the p-value retrieved by the hypergeometric test for 10 expressed miRNAs, from which a certain amount are also active. **a, b** human dataset. **c, d** Mouse dataset. **a, c** Black vertical lines represent active miRNAs. **b, d** Black vertical lines represent non-active miRNAs. Full computation details can be found in Methods.

Single-cell miRNA expression vs. activity plots

The plots in Supplementary Fig. 8 were produces for both mice and humans datasets in the following manner. First, a list of active miRNAs was generated, including all miRNAs with an average activity p-value less than 0.05 across all cells. Another list was generated in which miRNAs were ordered according to their total expression levels across all cells, arranged from the most expressed to the least expressed. The algorithm iterated over the expressed miRNAs list and produced a plot if the following criteria were met: 1. A matching miRNA was found in the active miRNA list, either as is or with 5p or 3p strands. 2. At least 10 cells remained after removing cells with expression = 0, and cells with expression greater than or equal to the 0.995 quantile. The algorithm generated plots for the first 21 miRNAs that matched the criteria for the human dataset, and 9 plots for the mouse dataset (since no other miRNA matched the criteria). Also, Pearson correlation coefficients were computed to evaluate the agreement between expression and activity on a cell level. Full results can be found at `https://doi.org/10.5281/zenodo.10720979`.

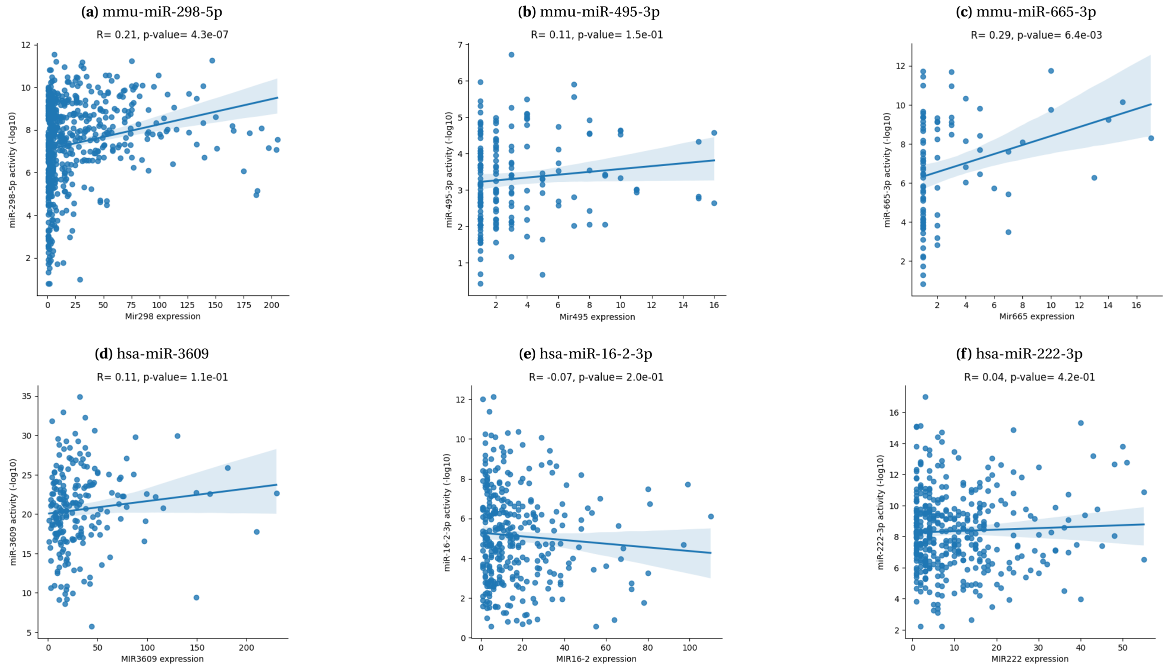

Supplementary Fig. 8: Single-cell miRNA expression vs. activity correlation plots. Top three most expressed and adequately active miRNAs from the mouse dataset are displayed in **a-c**, as well as top three miRNAs from the human dataset in **d-f**. Non-expressed cells and outliers were excluded. Data was obtained from[16]. More plots can be found at `https://doi.org/10.5281/zenodo.10720979`.

A mathematical simulation of a universally active miRNA

We simulated a dataset of 1,000 cells and 10,000 genes. We then simulated a miRNA with randomly chosen 100 targets. We randomly sampled expression values: for the nontarget genes, from a log-normal distribution with $\mu = 0$ and $\sigma = 0.5$, and for the target genes, from a log-normal distribution with $\mu = 0$ and $\sigma = 2$. We then applied miTEA-HiRes on the count matrix and found that the simulated miRNA is almost universally active (specifically, 993 cells had an activity p-value under 0.05- see Supplementary Fig. 9).

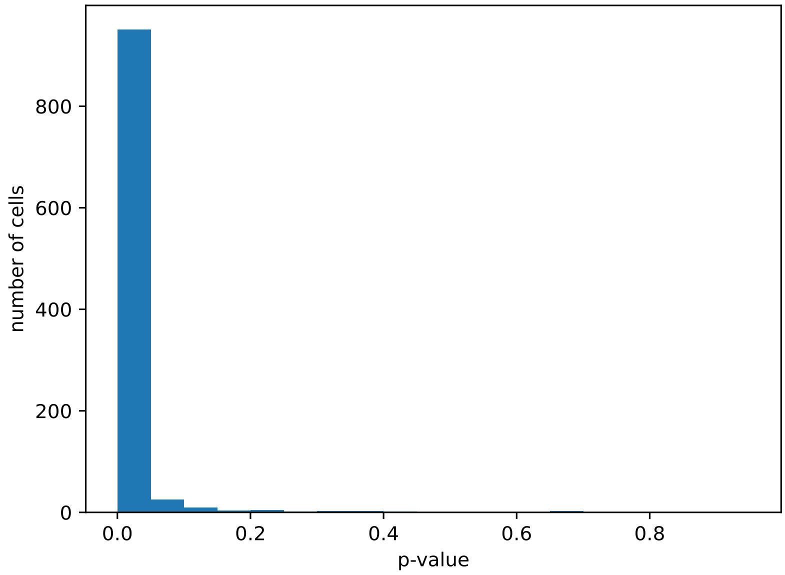

(a)

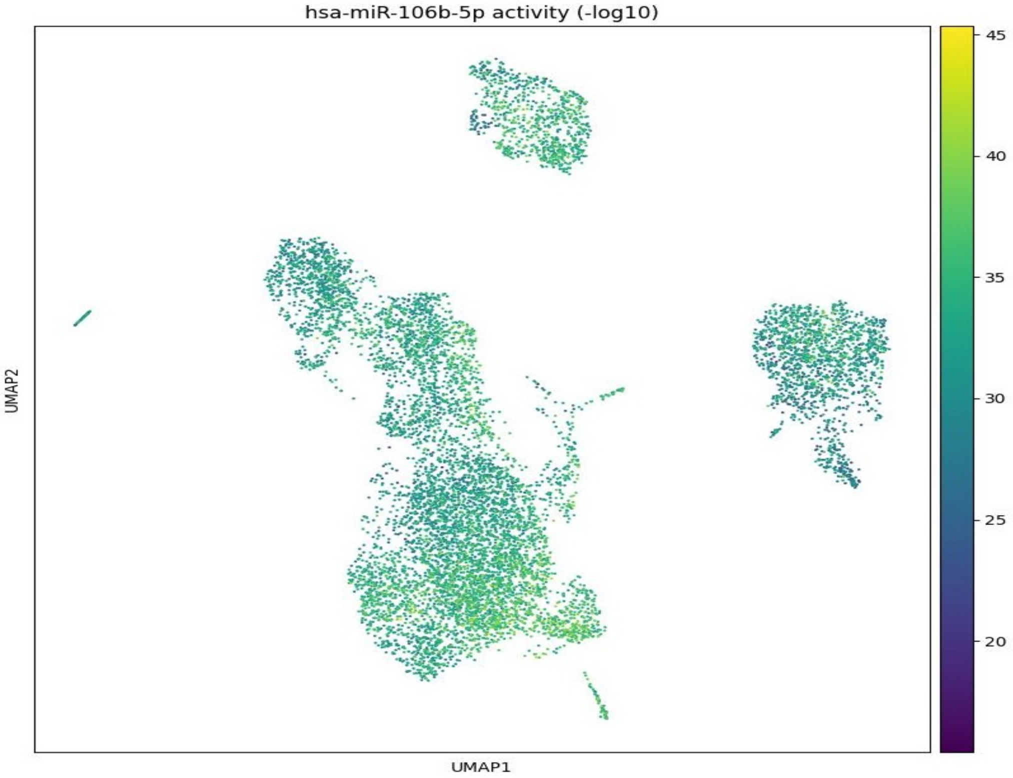

(b)

Supplementary Fig. 9: a Histogram of activity p-values in a simulated dataset. **b** Activity map of miR-106b-5p in the MS PBMCs dataset.

Example for gene expression based clustering compared to activity p-value distribution

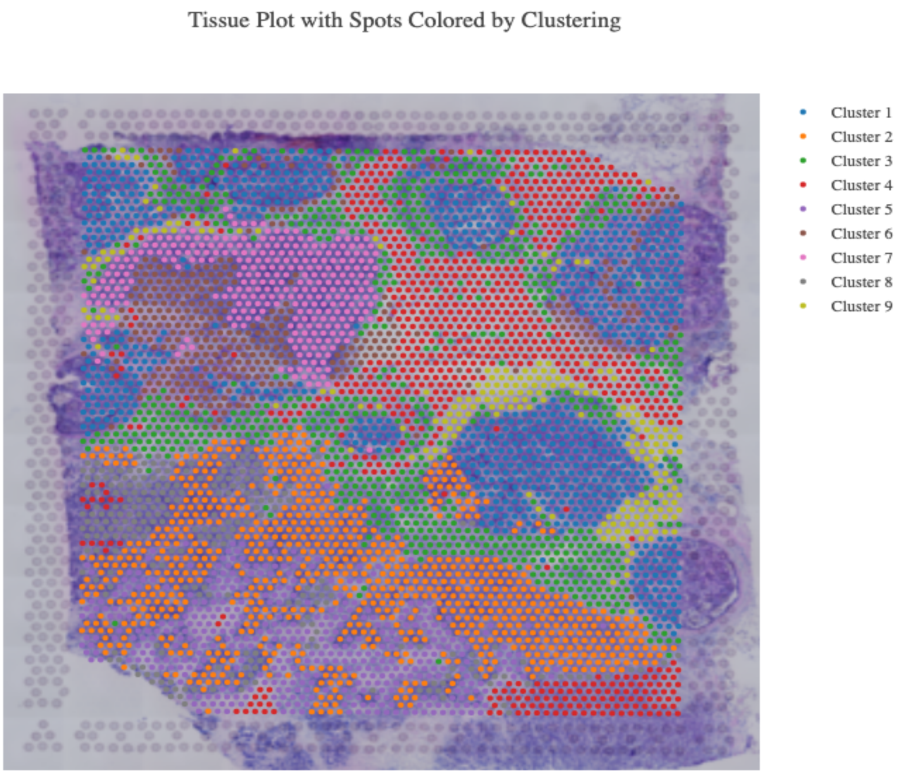

(a)

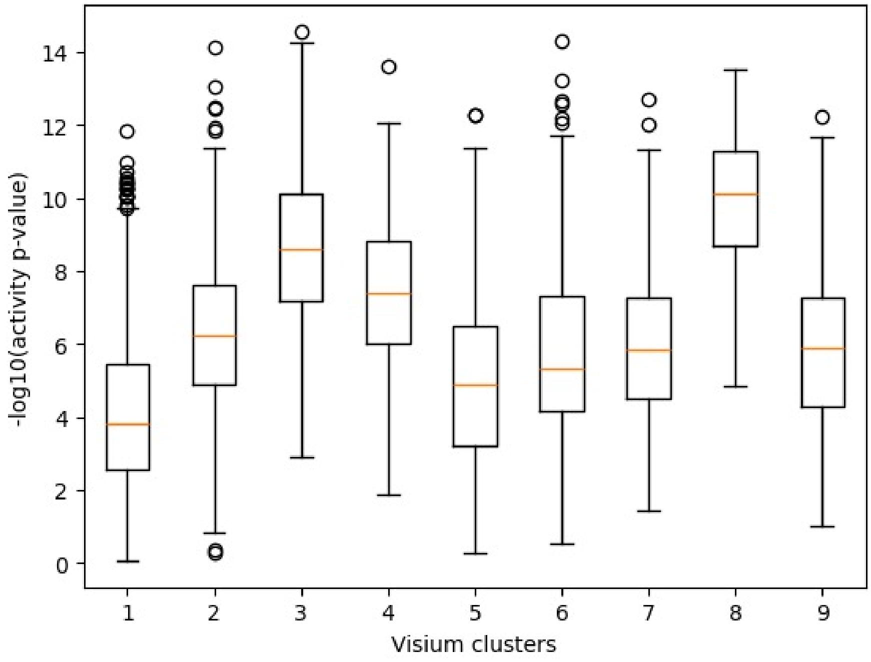

(b)

Supplementary Fig. 10: Comparison between **a** gene expression based clustering in human breast cancer[1] (see Figure 2), and **b** corresponding miR-29c-3p activity p-value distribution (-log10 transformed) obtained by miTEA-HiRes.

Comparison of target expression between conditions in the MS dataset

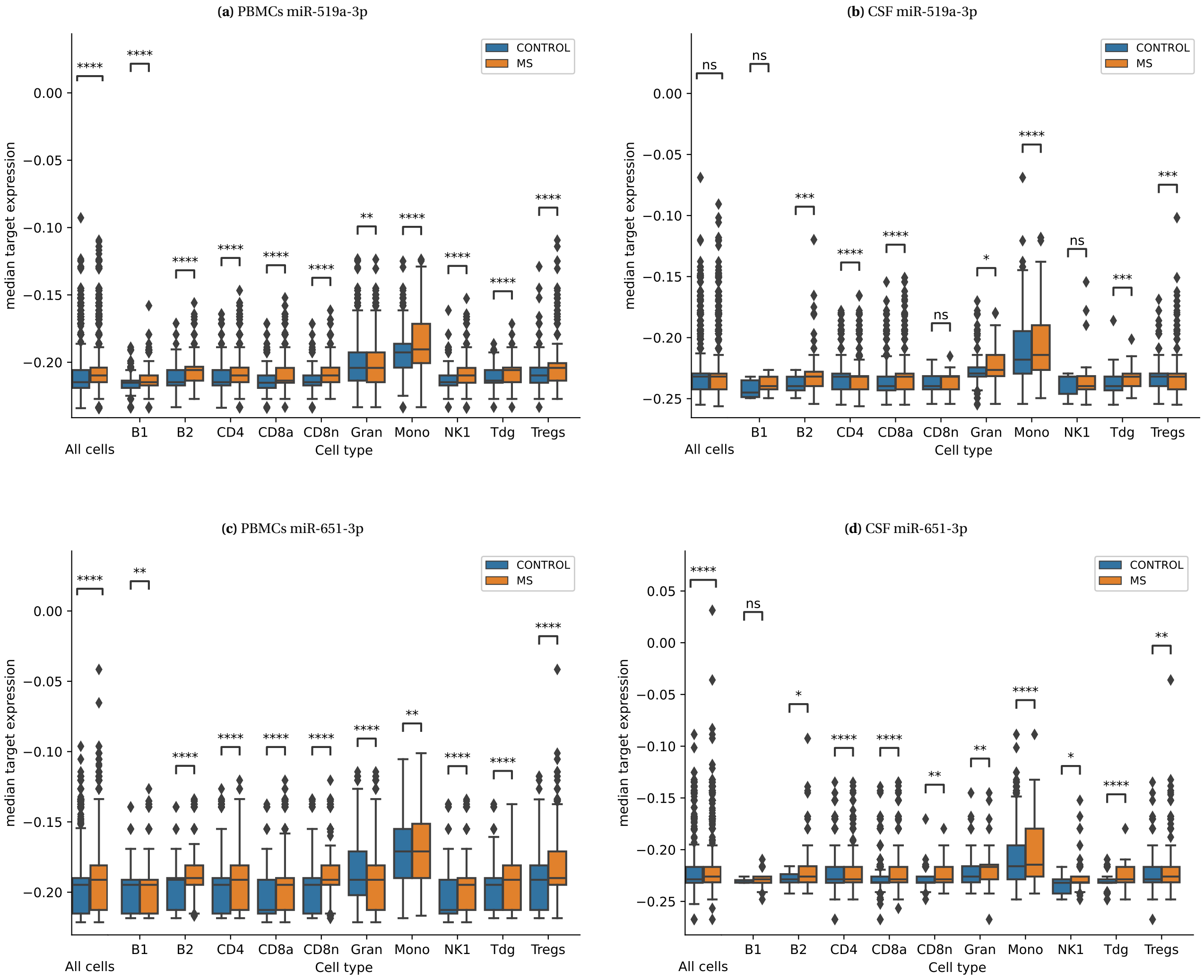

Supplementary Fig. 11: Box plots indicating difference in target expression between conditions compared in Figure 3. For each cell, "median target expression" was calculated as the median of Z-scored normalized expression values of the miRNA targets. **a** miR-519a-3p analysis on PBMCs; **b** miR-519a-3p in CSF cells; **c** miR-651-3p in PBMCs; **d** miR-651-3p in CSF cells. Two sided WRS test p-value legend: ns: $p > 0.05$; * : $0.01 < p \leq 0.05$; ** : $1e-3 < p \leq 0.01$; *** : $1e-4 < p \leq 1e-3$. **** : $p \leq 1e-4$. Cell-type keys: B1 and B2: B cell subsets; CD4: CD4+ T cells; CD8a: activated CD8+ T cells; CD8n: non-activated CD8+ T cells; Gran: granulocites; Mono: monocytes; NK1: natural killer cells; Tdg: $\gamma\delta$ T cells; Tregs: regulatory CD4+ T cells[5]. Group sizes can be found in Supplementary Table 4.

Spatial miRNA activity maps for downsampled gene expression data

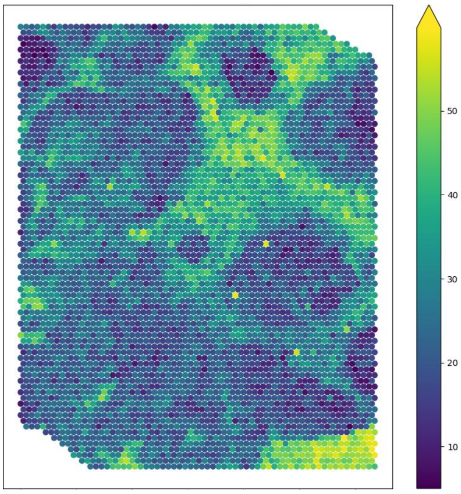

(a) Without downsampling

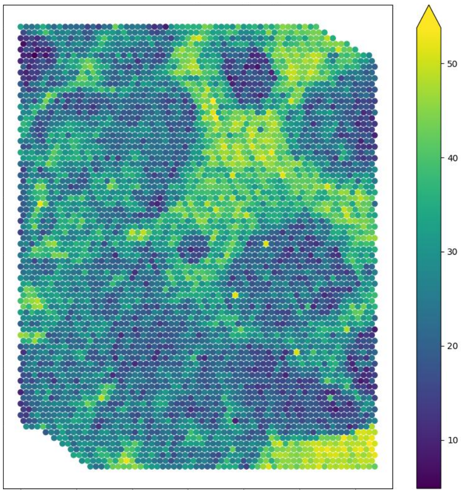

(b) All genes downsampled

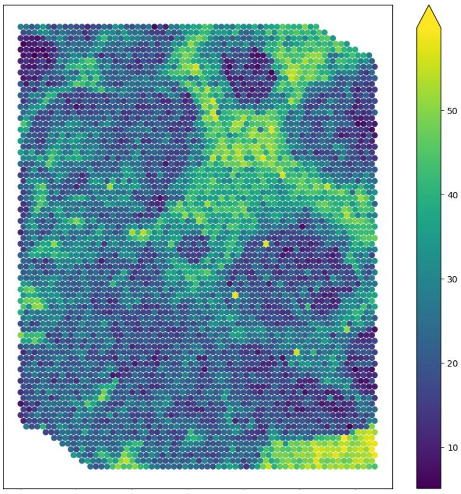

(c) Target genes downsampled

Supplementary Fig. 12: miR-16-5p spatial activity maps, as produced by miTEA-HiRES for Visium breast cancer spatial dataset. **a** original dataset. **b** after downsampling all the genes. **c** after downsampling only miR-16-5p's target genes. Downsampling was performed such that each UMI had 0.5 probability to be retained or set to 0 for each spot for each downsampled gene. Color bar represent -log10(activity p-value). The dataset has 4,898 spots.

Supplementary References

1. 10x Genomics. *Human Breast Cancer* `https://www.10xgenomics.com/resources/datasets/human-breast-cancer-visium-fresh-frozen-whole-transcriptome-1-standard`. Accessed: September 2022.
2. 10x Genomics. *Human Skin Melanoma (CytAssist FFPE)* `https://www.10xgenomics.com/resources/datasets/human-melanoma-if-stained-ffpe-2-standard`. Accessed: September 2022.
3. 10x Genomics. *Human Lung Squamous Cell Carcinoma (CytAssist FFPE)* `https://www.10xgenomics.com/resources/datasets/human-lung-cancer-ffpe-2-standard`. Accessed: September 2022.
4. 10x Genomics. *Human Ovarian Carcinoma (CytAssist FFPE)* `https://www.10xgenomics.com/resources/datasets/human-ovarian-cancer-11-mm-capture-area-ffpe-2-standard`. Accessed: September 2022.
5. Schafflick, D. *et al.* Integrated single cell analysis of blood and cerebrospinal fluid leukocytes in multiple sclerosis. *Nature communications* **11,** 247 (2020).
6. Luo, D. *et al.* Identification and functional analysis of specific MS risk miRNAs and their target genes. *Multiple Sclerosis and Related Disorders* **41,** 102044 (2020).
7. Shafiei, J., Javadi, G., Nateghi, B., Shaygannejad, V. & Salehi, M. Up-regulation of circulating miR-93-5p in patients with relapsing-remitting multiple sclerosis. *Journal of Basic Research in Medical Sciences* **6,** 4–11 (2019).
8. Tan, W., Li, Y., Lim, S.-G. & Tan, T. M. miR-106b-25/miR-17-92 clusters: polycistrons with oncogenic roles in hepatocellular carcinoma. *World Journal of Gastroenterology: WJG* **20,** 5962 (2014).
9. Sağır, F. *et al.* miR-132-3p, miR-106b-5p, and miR-19b-3p Are associated with brain-derived neurotrophic factor production and clinical activity in multiple sclerosis: a pilot study. *Genetic testing and molecular biomarkers* **25,** 720–726 (2021).
10. Hecker, M. *et al.* MicroRNA expression changes during interferon-beta treatment in the peripheral blood of multiple sclerosis patients. *International journal of molecular sciences* **14,** 16087–16110 (2013).
11. Landais, S., Landry, S., Legault, P. & Rassart, E. Oncogenic potential of the miR-106-363 cluster and its implication in human T-cell leukemia. *Cancer research* **67,** 5699–5707 (2007).
12. Mogilyansky, E. & Rigoutsos, I. The miR-17/92 cluster: a comprehensive update on its genomics, genetics, functions and increasingly important and numerous roles in health and disease. *Cell Death & Differentiation* **20,** 1603–1614 (2013).
13. Maciak, K., Dziedzic, A., Miller, E. & Saluk-Bijak, J. miR-155 as an important regulator of multiple sclerosis pathogenesis. A review. *International journal of molecular sciences* **22,** 4332 (2021).
14. Martinez, B. & Peplow, P. V. MicroRNAs in blood and cerebrospinal fluid as diagnostic biomarkers of multiple sclerosis and to monitor disease progression. *Neural regeneration research* **15,** 606 (2020).
15. Cox, M. B. *et al.* MicroRNAs miR-17 and miR-20a inhibit T cell activation genes and are underexpressed in MS whole blood. *PloS one* **5,** e12132 (2010).
16. Isakova, A., Neff, N. & Quake, S. R. Single-cell quantification of a broad RNA spectrum reveals unique noncoding patterns associated with cell types and states. *Proceedings of the National Academy of Sciences* **118,** e2113568118 (2021).
