# Supplementary material for: Inferring single-cell and spatial microRNA activity from transcriptomics data
Source: Commun Biol. 2025 Jan 18;8:87. doi: 10.1038/s42003-025-07454-9 (PMC11743151; doi:10.1038/s42003-025-07454-9)
Supplement: Supplementary file 3 — Reporting Summary [file 42003_2025_7454_MOESM3_ESM.pdf]

nature portfolio

Corresponding author(s): Efrat Herbst

Last updated by author(s): Sep 2, 2024

Reporting Summary

Nature Portfolio wishes to improve the reproducibility of the work that we publish. This form provides structure for consistency and transparency in reporting. For further information on Nature Portfolio policies, see our Editorial Policies and the Editorial Policy Checklist.

Statistics

For all statistical analyses, confirm that the following items are present in the figure legend, table legend, main text, or Methods section.

n/a	Confirmed	
☐	☒	The exact sample size (*n*) for each experimental group/condition, given as a discrete number and unit of measurement
☐	☒	A statement on whether measurements were taken from distinct samples or whether the same sample was measured repeatedly
☐	☒	The statistical test(s) used AND whether they are one- or two-sided<br>*Only common tests should be described solely by name; describe more complex techniques in the Methods section.*
☒	☐	A description of all covariates tested
☐	☒	A description of any assumptions or corrections, such as tests of normality and adjustment for multiple comparisons
☐	☒	A full description of the statistical parameters including central tendency (e.g. means) or other basic estimates (e.g. regression coefficient) AND variation (e.g. standard deviation) or associated estimates of uncertainty (e.g. confidence intervals)
☐	☒	For null hypothesis testing, the test statistic (e.g. *F*, *t*, *r*) with confidence intervals, effect sizes, degrees of freedom and *P* value noted<br>*Give P values as exact values whenever suitable.*
☒	☐	For Bayesian analysis, information on the choice of priors and Markov chain Monte Carlo settings
☒	☐	For hierarchical and complex designs, identification of the appropriate level for tests and full reporting of outcomes
☐	☒	Estimates of effect sizes (e.g. Cohen's *d*, Pearson's *r*), indicating how they were calculated

Our web collection on statistics for biologists contains articles on many of the points above.

Software and code

Policy information about availability of computer code

Data collection	No software was used.
Data analysis	Our custom code is available as python library: mitea_hires==0.0.19.<br>Python libraries used: anndata==0.8.0, numpy==1.23, pandas==1.5.2, scanpy==1.9.1, scipy==1.8.1 and xlmhg==2.5.4.

For manuscripts utilizing custom algorithms or software that are central to the research but not yet described in published literature, software must be made available to editors and reviewers. We strongly encourage code deposition in a community repository (e.g. GitHub). See the Nature Portfolio guidelines for submitting code & software for further information.

Data

Policy information about availability of data

All manuscripts must include a data availability statement. This statement should provide the following information, where applicable:

- Accession codes, unique identifiers, or web links for publicly available datasets
- A description of any restrictions on data availability
- For clinical datasets or third party data, please ensure that the statement adheres to our policy

Spatial transcriptomics read counts and spatial coordinates were downloaded from the Visium website (https://www.10xgenomics.com/products/spatial-gene-expression) for the following tissues:
1.Mouse brain (https://www.10xgenomics.com/resources/datasets/adult-mouse-brain-ffpe-1-standard-1-3-0).

2. Human breast cancer (https://www.10xgenomics.com/resources/datasets/human-breast-cancer-visium-fresh-frozen-whole-transcriptome-1-standard).
3. Human skin melanoma (https://www.10xgenomics.com/resources/datasets/human-melanoma-if-stained-ffpe-2-standard).
4. Human lung cancer (https://www.10xgenomics.com/resources/datasets/human-lung-cancer-ffpe-2-standard).
5. Human ovarian cancer (https://www.10xgenomics.com/resources/datasets/human-ovarian-cancer-11-mm-capture-area-ffpe-2-standard).
6. Human cerebellum (https://www.10xgenomics.com/resources/datasets/human-cerebellum-targeted-neuroscience-panel-1-standard).

The Multiple Sclerosis (MS) raw count matrices were downloaded from GEO repository under the accession number: GSE138266 (https://www.ncbi.nlm.nih.gov/geo/query/acc.cgi?acc=GSE138266).
The MS processed count matrices, with cell type annotations, were downloaded from: https://github.com/chenlingantelope/MSscRNAseq2019.git.

The breast cancer migratory and stationary count matrices were downloaded from GEO repository under the accession number: GSE162726 (https://www.ncbi.nlm.nih.gov/geo/query/acc.cgi?acc=GSE162726).

The totalRNA human and mouse datasets were downloaded from the GEO repository under the accession number: GSE151334 (https://www.ncbi.nlm.nih.gov/geo/query/acc.cgi?acc=GSE151334).

The count matrices of the miRNA induction experiments that were used for validation, were obtained directly from the authors Rzepiela, A. J.

Bulk cancer data that was used for validation was downloaded from the GDC portal: https://gdc.cancer.gov/

Research involving human participants, their data, or biological material

Policy information about studies with human participants or human data. See also policy information about sex, gender (identity/presentation), and sexual orientation and race, ethnicity and racism.

Reporting on sex and gender	N/A because our research used only published datasets.
Reporting on race, ethnicity, or other socially relevant groupings	N/A because our research used only published datasets.
Population characteristics	N/A because our research used only published datasets.
Recruitment	N/A because our research used only published datasets.
Ethics oversight	N/A because our research used only published datasets.

Note that full information on the approval of the study protocol must also be provided in the manuscript.

Field-specific reporting

Please select the one below that is the best fit for your research. If you are not sure, read the appropriate sections before making your selection.

☒ Life sciences ☐ Behavioural & social sciences ☐ Ecological, evolutionary & environmental sciences

For a reference copy of the document with all sections, see nature.com/documents/nr-reporting-summary-flat.pdf

Life sciences study design

All studies must disclose on these points even when the disclosure is negative.

Sample size	We showed usability of our algorithm using previously published datasets:<br>Spatial transcriptomics (Visium) - all spots in the datasets were taken into account.<br>Multiple Sclerosis: randomly sampled cells, as explained below.<br>Multiple Sclerosis annotated dataset - all cells were taken into account.<br>Single cell breast cancer: all cells were taken into account.<br>TotalRNA: for computations of association between expression and activity of miRNAs we aggregated all cells. For computations of correlations shown in supplementary fig. 5, we used all non-outlier cells expressing the relevant miRNA.<br>Single-cell mRNA of miRNA induction experiments: all cells were taken into account as described below.<br>Bulk mRNA-miRNA data used for validation: We established a list of 11,927 cases in the GDC portal that have both bulk RNAseq sample and a miRNAseq sample, from a solid tissue primary tumor. The samples had to be open access and in one of the formats tsv or txt.
Data exclusions	To reduce computation time:<br>Multiple Sclerosis PBMCs: 10K cells were randomly sampled.<br>Multiple Sclerosis CSF cells: 10K cells were randomly sampled.<br>Supplementary fig. 5: cells with miRNA expression = 0 were excluded, as well as cells with miRNA expression >= 0.995 quantile.<br>In all single-cell datasets, duplicated cells with identical gene expression profiles were removed.<br>Single-cell mRNA of miRNA induction experiments: cells with less than 5,000 detected genes were excluded, as well as cells with gene detection rate > 0.98 quantile. This step was done because we noted that one of the datasets had substantially lower gene detection rate.<br>Bulk mRNA-miRNA: if more than one sample was available for a patient, the one with highest read was selected.

Replication	Software was executed multiple times obtaining similar results.
Randomization	This is not relevant because we used published datasets.
Blinding	This is not relevant because we used published datasets.

Reporting for specific materials, systems and methods

We require information from authors about some types of materials, experimental systems and methods used in many studies. Here, indicate whether each material, system or method listed is relevant to your study. If you are not sure if a list item applies to your research, read the appropriate section before selecting a response.

Materials & experimental systems

n/a	Involved in the study
☒	☐ Antibodies
☒	☐ Eukaryotic cell lines
☒	☐ Palaeontology and archaeology
☒	☐ Animals and other organisms
☒	☐ Clinical data
☒	☐ Dual use research of concern
☒	☐ Plants

Methods

n/a	Involved in the study
☒	☐ ChIP-seq
☒	☐ Flow cytometry
☒	☐ MRI-based neuroimaging

Plants

Seed stocks	N/A because no plants were used in our study.
Novel plant genotypes	N/A because no plants were used in our study.
Authentication	N/A because no plants were used in our study.
