# Supplementary material for: Inferring single-cell and spatial microRNA activity from transcriptomics data
Source: Commun Biol. 2025 Jan 18;8:87. doi: 10.1038/s42003-025-07454-9 (PMC11743151; doi:10.1038/s42003-025-07454-9)
Supplement: Supplementary file 1 — Transparent Peer Review file [file 42003_2025_7454_MOESM1_ESM.pdf]

nature portfolio

Peer Review File

Inferring single-cell and spatial microRNA activity from transcriptomics data

Corresponding Author: Ms Efrat Herbst

This file contains all reviewer reports in order by version, followed by all author rebuttals in order by version.

Version 0:

Reviewer comments:

Reviewer #1

(Remarks to the Author)
The manuscript introduces a computational method, miTEA-HiRes, for inferring miRNA activity from single-cell and spatial transcriptomics data using the expression level of known targets of miRNAs. An active miRNA reduces the expression of the target genes, and miTEA-HiRes uses minimum hypergeometric test to identify significantly low expression of target genes. miTEA-HiRes was applied to multiple single-cell and spatial transcriptomimcs datasets and revealed known oncogenic miRNAs in tumor samples.

However, my main concern is that the manuscript does not contain any evaluation of the accuracy of the inferred activity or comparisons with previous computational methods. Without evaluating the accuracy of the proposed method, I am not convinced whether or not the conclusions in analyzing real data are reliable.

Additionally, I have the following minor questions/suggestions:
1. The minimum hypergeometric test requires that the number of target genes is not too small (e.g., only 1 target gene). It would be helpful to include some summary statistics of the miRNA-target gene information, including how many miRNAs, on average how many target genes each miRNA regulates.
2. The choice of when to p-value correction is a little confusing and not well justified. According to the current description of the second step, it seems that the aggregated activity score in spatial transcriptomics data is by threshold un-adjusted p-values, while the aggregation for single-cell data is based on corrected p-values.
3. The three terms, “Score", “p-value”, and adjusted p-value, need more clear definitions and to be used more consistently across the manuscript.
4. The analysis includes comparing the activity across different patients and different conditions. But different patients and conditions will likely have batch effects. Is miTEA-HiRes robust to batch effect? I suggest adding simulated data to demonstrate this point.

Reviewer #2

(Remarks to the Author)
Herbst et al. have developed a statistical method called miTEA-HiRes to predict miRNA activity in single-cell and spatial transcriptomics data. The algorithm is based on their previous method, miTEA, which predicts miRNA activity in bulk samples. The underlying assumption is that if a specific miRNA is active, its target genes should be down-regulated, and hence miRNA activity can be inferred from its targets’ expression. The score for miRNA activity is based on the minimal hyper-geometric test for the enrichment of the miRNA targets in the sorted vector of highly expressed genes in each cell or spot. The authors have used miTEA-HiRes to analyze miRNA activity in various public datasets, including cancer and brain spatial expression, single-cell RNA-seq data of PBMCs and CSF from MS patients, and single-cell RNA-seq data of cancer cells.

Measuring miRNA expression at the single-cell level is currently a difficult task using existing experimental methods. Thus, miTEA-HiRes presents an opportunity to explore miRNA involvement and regulation in single-cell resolution. However, my main concern is regarding the second step of the algorithm. The first step calculates an activity p-value for each miRNA in each cell or spot. In the second step, these p-values are averaged across all cells to produce an activity score for each miRNA for the entire sample. The problem with averaging the activity scores across all different cells is that we lose the

heterogeneity of the single-cell data we wanted to capture, and instead, we end up using the data as pseudo-bulk. Furthermore, this step is highly sensitive to the sample composition, such as the composition of immune cells in PBMCs or malignant cells in cancer tissue, as the impact of larger populations on the averaged results is more pronounced. Therefore, to gain more insights into miRNA activity in different cell types, subtypes, and disease states, I would suggest performing the analysis for each cell/spot separately (as in step 1 of the algorithm) and clustering the data based on these activity scores. Exploring the active miRNAs in each cluster and associating these clusters to the different cell types and states can shed light on miRNA involvement in health and disease in different cell types.

Another mode of the algorithm enables the comparison of activity scores between two groups, such as healthy individuals and patients or different cell types. I suggest modifying this mode to allow for the comparison of multiple groups, enabling comparison between all the different cell types within the sample.

Major concerns:

1) Figure 2 – It would be helpful to add a boxplot of the p-values activity scores of each spot in the different GE clusters to statistically evaluate whether specific miRNA activity is associated with specific clusters. Otherwise, it is hard to evaluate the author's statement, "For example, the activity of miR-29c-3p in the breast cancer tissue (Fig. 2, second row) does not precisely correspond to either the clustering patterns or the characteristics observed in the H&E slide." (lines 159-161).

2) In Figure 2, the activity scores of miR-16-5p and miR-29c-3p appear similar. However, it is crucial to consider whether they share the same targets. Overlapping targets of different miRNAs can significantly influence the analysis results. In some cases, two miRNAs may have similar targets, leading to similar activity scores, even if only one of them is active. I suggest classifying all miRNAs based on their target similarity to explore potential overlaps between miRNAs. Additionally, it is important to notify the user about such miRNAs to ensure accuracy in the analysis.

3) PBMCs analysis – Averaging the activity p-values across all cells of the sample can make it difficult to interpret biological insight, as the sample contains different cell types with different miRNA activity. Moreover, different activity between two groups (such as healthy and MS) could arise from varying compositions of their PBMCs. For instance, if one sample has 80% T cells and the other has only 30% T cells (a variability that exists in healthy populations), a miRNA that is active in T cells in general will likely be significant only in the sample with the higher percentage of T cells, irrespective of any change in regulation in MS patients. The strength and novelty of this method lie in its application to single-cell data. However, averaging all cells of the sample transforms it into a pseudo-bulk dataset, thereby losing the heterogeneity of the single-cell sample.
Additionally, averaging the scores on healthy and MS patients together is even less clear, and it may be better to remove this mode or change it to be only on the MS PBMCs.

4) Line 190 – Are the selected 10,000 cells equally distributed among healthy and MS patients? If not, it might affect the results.

5) Figure 3: Some miRNAs show a significant difference in activity between control and MS samples. However, based on their p-values, they are also significant in each condition alone. What does it mean when there is a difference in activity between conditions if the miRNA is active in both conditions? Could it be due to different targets, resulting in different scores? If so, it would be interesting to investigate. Can you check for the targets that are associated with each condition? Specifically, those that are enriched in the mGH test of each condition.

Additionally, it is important to specify the number of cells from each population since, in some comparisons, it seems that the difference between populations is insignificant due to the number of cells and not due to lack of difference (for example, miR-519a-3p B1 population in the CSF data).

6) Table 3 – Why is the comparison only between 2 cell types? Why not compare the activity scores across all cell types? I would add a possibility to compare the activity scores between multiple groups.

7) Figure 4: d-e seems significant between static and migratory cells only due to one dataset (SUM159). As a result, their histograms display a slight separation between static and migratory cells (h-i). To account for miRNAs that are significant in only one dataset, it will be worthwhile to analyze the effect of each dataset individually as well as collectively.

8) Section 2.5 –To rank the miRNAs by expression levels, the authors summed the raw counts of each miRNA across all the cells. This was likely done because single-cell data is often sparse. However, I'm not entirely sure why they chose to do this, as by averaging across all cells, we lose the variability of the single-cell data and mix signals from cells where the miRNA is active with cells where it is not. What is the advantage of performing this analysis over using bulk data?

9) Line 363 – If I understand correctly, you are using the data from HEK293T and MCF7 together as one sample, which introduces even more "noise" or heterogeneity. The same applies to the mouse data, where you are combining all stages of embryonic stem cell differentiation. Have you considered analyzing each dataset alone? It might lead to clearer insights.

10) Lines 407-414: This scenario can occur if the miRNA activity originates from a particular group within the sample with a strong signal that can influence the entire population. This highlights the importance of avoiding averaging activity scores

across all cells but instead focusing on specific groups of cells with similar characteristics. Doing so helps to preserve the unique attributes of individual cells in the dataset and avoids incorrect interpretations of the data.

Minor comments:

Figure 1: Change sample to cell, confusing.

Figure 2: The label of the color bar is very small and hard to read, in addition, it should be -log10(activity p-value) and not p-value(-log10). The labels in other figures are also very small (for example, Figure 4).

Figure 4 – In the text, it initially referred to figures 4h-k and later to figures 4f-g.

Figure 5: Only appears in the discussion part, not referred to in section 2.5

Table 1: Very hard to see the histograms of the p-values.
Why, for miR-19a-3p, is there no p-value and histogram for the comparative activity mode?

Lines 356 – 357 – The author can check their assumption using pathway enrichment of the specific targets of these miRNAs which are lowly expressed in this sample.

Reviewer #3

(Remarks to the Author)
Herbst et al. present an interesting approach to look at miRNA activity in single cell and spatial datasets, relying on the expression patterns of targets (with miRNA-target interaction supported by experimental evidence). The method is exemplified in multiple spatial and single cell transcriptomics datasets and results seem to match literature or provide novel insights for less studied miRNAs. By providing a github repository and pip install, the authors also facilitate other researchers to apply their approach to novel datasets.

I have a few comments, mainly regarding the method:
1) Line 498-511: Are differences in coverage taken into account during the preprocessing steps (e.g. by downsampling or by requiring a minimum number of reads)? In case there are both cells with few reads and cells with considerably more reads, the counts of (the fewer) detected genes in the first may be inflated after normalization (which will impact the z-scores per gene).

2) The activity p-values are currently corrected for multiple testing, yet there are many tests performed in one dataset analysis. The provided p-values may as such be overoptimistic and a multiple testing correction or at least a remark may be appropriate. For the comparitive activity mode scores, the authors do use FDR correction and they also prioritize smaller activity p-values later in the paper.

3) The activity score calculation is not entirely clear to me. For the single cell data, this is an adjusted p-value (average or based on Wilcoxon rank-sum test) where a smaller activity score indicates miRNA activity. For spatial data, however, it is explained as the percentage of spots that has an activity p-value below a certain threshold. In this case, a higher activity score seems to indicate miRNA activity. Is this correct? Can this be further explained in the methods section?

4) On line 222, the authors deviate from the default of 10k cells. How was the number of cells in general determined and can the authors provide guidelines for other users about the optimal number of cells for a particular analysis?

5) Supplementary Fig. 4 is not entirely clear for me. A & b are from one dataset, c&d from another, with black lines representing either the active or non-active miRNAs. Taking into account the different ranking (descending in a&c, ascending in b & d) and thus mirroring effect, the black bars still do not replace all white space and vice versa when comparing a&b or c&d (active vs inactive miRNAs). Should this not be the case?

6) Minor comment: some typos in the Github readme (e.g. comPArative activity, traNscriptomics, compuTation)

Version 1:

Reviewer comments:

Reviewer #1

(Remarks to the Author)
My previous concerns were addressed in this revision. The manuscript reads much more clearly to me. With more understanding, I have the following comments.

1. P-values are compared using Wilcoxon rank sum test in the “comparative activity mode”. But I think the “effect sizes” is usually the focus of comparisons, I.e., given two groups (A and B) and a miRNA, the most direct question is whether the target genes in group A are of lower expression than those target genes in group B. Does Wilcoxon rank sum test of p-values lead to the same conclusion as using Wilcoxon rank sum test on the target genes between the two groups? Or can this point be at least discussed?
2. Another question about p-values is that they are averaged in the “total activity mode”. But there are many other ways to combine p-values, such as Fisher's method (which derives the expected distribution when summing up the log of p-values) or Cauchy combination rule. Why using average rather than other p-value aggregation methods? And why averaging p-values rather than log-transformed p-values?
3. Following the comment of reviewer 2 in the previous review, the statement “For example, the activity of miR-29c-3p in the breast cancer sample (Fig. 2, second row) does not precisely correspond, at the qualitative visual inspection level, to either the clustering patterns or the characteristics observed in the H&E slide” in line 175—177 is somewhat suspicious in my view, as the activity map looks like a blurry version of that of miR-16-5p. As spatial Transcriptomics data is sparse and the ranking of gene expression may be affected by random variation, I wonder if the target genes of a miRNA in general have low expression and a high percentage of zeros across the spots, can the activity map be accurately identified? A potential experiment is to down-sample the counts of target genes related to miR-16-5p (e.g. each UMI has 0.5 probability to retained or set to 0 for each spot for each target gene) and infer the activity map, whether the activity map is visually consistent with the original one before down-sampling.
4. The notation Λ in equation (1) after line 624 doesn’t seem to be defined.

Reviewer #2

(Remarks to the Author)
In this revision, the authors have primarily made changes to the text rather than introducing new analyses. While this approach is acceptable in most instances, there are specific cases where incorporating the requested analyses would significantly enhance the quality of the paper and its conclusions.

1. For Figure 2, a straightforward analysis of adding a boxplot of the p-values activity scores of each spot in the different GE clusters. This would help evaluate whether a specific miRNA activity is associated with a particular cluster.

2. I am still unclear about the meaning of the miRNAs that show differing activity between control and MS samples (Figure 3), while they are also significant in each condition alone. Further clarification is needed. I understand that you could not achieve statistical significance, but it would be beneficial to display the expression levels of the target genes in both control and MS cells to better understand this difference. Is there a variation in target expression between the two groups?

Reviewer #3

(Remarks to the Author)
The authors have carefully considered my comments from the previous review and have successfully addressed them in the revised manuscript. In particular, they have provided clarifications and added appropriate warnings in the text where necessary.

Version 2:

Reviewer comments:

Reviewer #1

(Remarks to the Author)
All my previous comments have been addressed in this revision.

Reviewer #2

(Remarks to the Author)
The authors have successfully addressed all of my previous suggestions and comments and incorporated the requested revisions in the updated manuscript

Open Access This Peer Review File is licensed under a Creative Commons Attribution 4.0 International License, which permits use, sharing, adaptation, distribution and reproduction in any medium or format, as long as you give appropriate credit to the original author(s) and the source, provide a link to the Creative Commons license, and indicate if changes were made.
In cases where reviewers are anonymous, credit should be given to 'Anonymous Referee' and the source.
The images or other third party material in this Peer Review File are included in the article's Creative Commons license, unless indicated otherwise in a credit line to the material. If material is not included in the article's Creative Commons license and your intended use is not permitted by statutory regulation or exceeds the permitted use, you will need to obtain permission directly from the copyright holder.
To view a copy of this license, visit https://creativecommons.org/licenses/by/4.0/

Comment	Response
Reviewer #1	
The manuscript introduces a computational method, miTEA-HiRes, for inferring miRNA activity from single-cell and spatial transcriptomics data using the expression level of known targets of miRNAs. An active miRNA reduces the expression of the target genes, and miTEA-HiRes uses minimum hypergeometric test to identify significantly low expression of target genes. miTEA-HiRes was applied to multiple single-cell and spatial transcriptomimcs datasets and revealed known oncogenic miRNAs in tumor samples.	We thank the reviewer for the summary and for the careful and useful review. We specifically appreciate the comments related to comparison to other software tools and statistical methods.
1. However, my main concern is that the manuscript does not contain any evaluation of the accuracy of the inferred activity or comparisons with previous computational methods. Without evaluating the accuracy of the proposed method, I am not convinced whether or not the conclusions in analyzing real data are reliable.	There are two separate issues here. One is the validation and one is the comparison.<br>As for validation, the only reasonable approach to assessing accuracy is to align our miR activity results with miR expression results, on the same or similar samples. As detailed below, we added analysis to this end.<br>As for comparison - we added analysis describing comparative studies working with miREACT and miRSCAPE.<br>In particular, we have added a new section "Validation and comparison to other methods" (see Section 3, rows 390-455 and Fig. 5) containing the following:<br>● Section 3.1. includes analysis of matched bulk RNA-miRNA data obtained from GDC. We perform a comparison of inferred miR activity to miR expression and a comparison to miREACT. We also present some anecdotal examples of miRNA activity profiles corresponding to their known function in specific cancer types.<br>● Section 3.2. Is based on text from the previous version, which we moved here, to better fit the context. In this section we assess accuracy by comparing activity to miRNA expression in two totalRNA datasets.<br>● Section 3.3. includes analysis of a single-cell miRNA induction experiment from Rzepiela et. al. We

Comment	Response
	use these data to assess accuracy and to compare to miRSCAPE and miREACT.
Additionally, I have the following minor questions/suggestions:<br>2. The minimum hypergeometric test requires that the number of target genes is not too small (e.g., only 1 target gene). It would be helpful to include some summary statistics of the miRNA-target gene information, including how many miRNAs, on average how many target genes each miRNA regulates.	Please find Supplementary Section 10.1 (rows 1171-1172) and Supplementary Fig. 1 including an additional histogram of the number of targets per miRNA (human and mouse), as well as summary statistics.
3. The choice of when to p-value correction is a little confusing and not well justified. According to the current description of the second step, it seems that the aggregated activity score in spatial transcriptomics data is by threshold un-adjusted p-values, while the aggregation for single-cell data is based on corrected p-values.	Individual activity p-values (representing the level of activity of a specific miRNA in a specific cell or spot) are not reported in the form of a list of significant results and therefore are not corrected. In activity maps (spatial or UMAPs), these activity p-values are indicated by the color.<br>On the second step of the algorithm, we aggregate these p-values, and this is done differently for spatial data and single-cell data.<br>For spatial datasets, we chose to order the miRNAs based on the percentage of active spots in the sample (in descending order), since we thought this is the best measure of activity across an entire slide. This measure is not a significance score and therefore correcting for multiple testing is irrelevant.<br>For single cell datasets in 'total activity' mode, we chose to order the miRNAs based on their average activity p-value over all cells, which we then corrected for multiple testing.<br>We understand that both measures may be used for both types of datasets, and we will include the option to choose which measure to use in the next implemented version of the miTEA-HiRes package. For now, users can perform whatever analysis they see fit, working with the raw pixel/sc results. The latter are conveniently and clearly reported in the current version.
4. The three terms, "Score", "p-value",	Thank you for this comment. We believe that

Comment	Response
and adjusted p-value, need more clear definitions and to be used more consistently across the manuscript.	the main confusion was with single cell datasets in 'comparative activity' mode, where we used the terms "corrected p-value" and "activity score" interchangeably. We added a comment about that in Section 2.1 (second step, lines 147-148). Added text: "For this mode, the terms "activity score" and "corrected WRS p-value" are used interchangeably."<br>We also made sure to use only the term "corrected WRS p-value" throughout the manuscript.
5. The analysis includes comparing the activity across different patients and different conditions. But different patients and conditions will likely have batch effects. Is miTEA-HiRes robust to batch effect? I suggest adding simulated data to demonstrate this point.	True, it is possible that in spite of the normalization procedure, batch effects still have some effect on the differential miRNA activity detected by miTEA-HiRes. To offset such effects the data can first be corrected for batch effect using a designated method (see Tran et al. 2020, Ben-Elazar et al. 2021), and then the corrected data can be used as input to miTEA-HiRes. We thank the reviewer for this comment and we also addressed this point in lines 117-120.
Reviewer #2	
Herbst et al. have developed a statistical method called miTEA-HiRes to predict miRNA activity in single-cell and spatial transcriptomics data. The algorithm is based on their previous method, miTEA, which predicts miRNA activity in bulk samples. The underlying assumption is that if a specific miRNA is active, its target genes should be down-regulated, and hence miRNA activity can be inferred from its targets' expression. The score for miRNA activity is based on the minimal hyper-geometric test for the enrichment of the miRNA targets in the sorted vector of highly expressed genes in each cell or spot. The authors have used miTEA-HiRes to analyze miRNA activity in various public datasets, including cancer and brain spatial expression, single-cell RNA-seq data of PBMCs and CSF from MS patients, and single-cell RNA-seq data of cancer cells. Measuring miRNA expression at the	Thank you for the comprehensive feedback. It is important to note the following. The inference of miR activity, through target enrichment analysis, does, indeed, facilitate the exploration of miRNA biology at a single-cell resolution, as a mere substitute for actual measured expression levels. This, however, is only one aspect of the scientific value of this analysis. Even if activity and expression are not so tightly linked (as other regulatory functions and elements may be involved in the process), the level of activity, in and of itself, can be an important determinant of regulation processes and of the biology of the cell.<br>We do see, however, the value of understanding the power of the miTEA approach as a proxy to expression levels and address the related points below.<br>We are grateful for the detailed and deep review and have followed many of the

Comment	Response
single-cell level is currently a difficult task using existing experimental methods. Thus, miTEA-HiRes presents an opportunity to explore miRNA involvement and regulation in single-cell resolution.	suggestions to add and modify text. Some others are excellent candidates for future projects.
1. However, my main concern is regarding the second step of the algorithm. The first step calculates an activity p-value for each miRNA in each cell or spot. In the second step, these p-values are averaged across all cells to produce an activity score for each miRNA for the entire sample. The problem with averaging the activity scores across all different cells is that we lose the heterogeneity of the single-cell data we wanted to capture, and instead, we end up using the data as pseudo-bulk. Furthermore, this step is highly sensitive to the sample composition, such as the composition of immune cells in PBMCs or malignant cells in cancer tissue, as the impact of larger populations on the averaged results is more pronounced. Therefore, to gain more insights into miRNA activity in different cell types, subtypes, and disease states, I would suggest performing the analysis for each cell/spot separately (as in step 1 of the algorithm) and clustering the data based on these activity scores. Exploring the active miRNAs in each cluster and associating these clusters to the different cell types and states can shed light on miRNA involvement in health and disease in different cell types.	This is an excellent point.<br>The output of the first step is an activity matrix, containing the activity p-values of every miRNA in every cell or spot analyzed. Users can use this matrix to perform further analysis, for example, as you suggested, clustering the cells based on these activity scores. More elaborate steps such as studying the activity specificity to cell types (as in Yanai, Itai, et al. "Genome-wide midrange transcription profiles reveal expression level relationships in human tissue specification." *Bioinformatics* 21.5 (2005): 650-659.) or other finer properties can also be performed. Future versions of miTEA-HiRes may also incorporate this direction suggested by the reviewer.<br>Furthermore - following this comment, we added text to Section 2.1 (see rows 128-130). The added text: “The activity matrix, containing the activity p-values, is provided as output for users to facilitate further user-defined analysis, in addition to the analysis performed by miTEA-HiRes pipeline. For example - an analysis based on clustering according to activity profiles can be performed. ”<br>Additionally, miTEA-HiRes outputs activity maps on a UMAP layout, where the activity p-values of the individual cells are depicted in color.<br>We chose to also apply the pseudo-bulk approach (averaging) in order to point the users to possible “generally-active” miRNAs. Of course, users may ignore this step and choose to work with individual activity maps. If cell type annotations are available, then the comparative activity mode can be invoked to investigate the miRNA activity heterogeneity in the sample (as we did with the MS dataset), as long as the groups are not too small for reliable statistical results.

Comment	Response
	Your concern that sample composition can affect averaged results is of course valid and also true in general for reported results on differentially expressed miRNAs in bulk data. We added Supplementary Table 4, depicting the fractions of different cell types in the MS dataset. As can be seen there, there is no major difference in the sample cell type composition between MS and control.
2. Another mode of the algorithm enables the comparison of activity scores between two groups, such as healthy individuals and patients or different cell types. I suggest modifying this mode to allow for the comparison of multiple groups, enabling comparison between all the different cell types within the sample.	Thank you for this suggestion, we plan to incorporate it in the next version of miTEA-HiRes.
Major concerns:<br>3. Figure 2 – It would be helpful to add a boxplot of the p-values activity scores of each spot in the different GE clusters to statistically evaluate whether specific miRNA activity is associated with specific clusters. Otherwise, it is hard to evaluate the author's statement, "For example, the activity of miR-29c-3p in the breast cancer tissue (Fig. 2, second row) does not precisely correspond to either the clustering patterns or the characteristics observed in the H&E slide." (lines 159-161).	We thank the reviewer for this important comment.<br>We agree that we don't provide sufficient statistical support to the orthogonality of miR activity and GE clusters. This can be a good topic for further investigation.<br>In the meantime we modified the specific text pointed out in the review, to clarify the qualitative nature of the observation, in lines 173-176, as follows:<br>"Other activity maps may offer a more unique insight- information about the tissue that cannot be inferred from the H&E or the GE clustering. For example, the activity of miR-29c-3p in the breast cancer sample (Fig. 2, second row) does not precisely correspond, at the qualitative visual inspection level, to either the clustering patterns or the characteristics observed in the H&E slide."
4. In Figure 2, the activity scores of miR-16-5p and miR-29c-3p appear similar. However, it is crucial to consider whether they share the same targets. Overlapping targets of different miRNAs can significantly influence the analysis results. In some	We agree that targets shared between miRNAs is an important consideration when evaluating results. We delved into the subject when we discussed miRNAs with similar activity maps in the breast cancer metastasis dataset (Results: lines 362-380, discussion: lines 497-503). We also agree that users

Comment	Response
cases, two miRNAs may have similar targets, leading to similar activity scores, even if only one of them is active. I suggest classifying all miRNAs based on their target similarity to explore potential overlaps between miRNAs. Additionally, it is important to notify the user about such miRNAs to ensure accuracy in the analysis.	should be informed of miRNAs with highly overlapping target lists, and it would definitely be incorporated into the next version of miTEA-HiRes.<br>For now, we computed the Jaccard index of overlap in target lists for all pairs of miRNAs and uploaded it to Zenodo.<br>We added a reference to this resource in the text (Results: lines 387-389, Discussion: lines 504-506 and code availability: lines 802-803).<br>Another option for users is to use miRPathDB to check the sequence similarity between pairs of miRNAs. miRNAs with similar sequences usually have similar targets.<br>As to miR-16-5p and miR-29c-3p, they do not have a significant overlap of target lists (Jaccard index: 0.026), so this is not the source of the similarity of their activity maps. The investigation of more miR pairs and the quantification of synergetic and/or antagonistic activity patterns, independent/dependent on sequence similarity, is a direction for continued work in our group.
5. PBMCs analysis – Averaging the activity p-values across all cells of the sample can make it difficult to interpret biological insight, as the sample contains different cell types with different miRNA activity. Moreover, different activity between two groups (such as healthy and MS) could arise from varying compositions of their PBMCs. For instance, if one sample has 80% T cells and the other has only 30% T cells (a variability that exists in healthy populations), a miRNA that is active in T cells in general will likely be significant only in the sample with the higher percentage of T cells, irrespective of any change in regulation in MS patients. The strength and novelty of this method lie in its application to single-cell data. However, averaging all cells of the sample transforms it into a pseudo-bulk dataset, thereby losing	We agree that changes in the compositions of compared groups may lead to false positive results (or the opposite, mask a true signal). For the MS dataset, we added the compositions of the MS and healthy groups as Supplementary Table 4, and it appears that their compositions are similar. However, in general, we offer users to use the miTEA-HiRes comparative activity mode to investigate whether the signal stems from difference in regulation or from specific cell types that present higher activity of some miRNA. If the cell types are unknown, users can still cluster their data and use the different clusters. We added a note about this in the discussion (lines 529-535).<br><br>miTEA-HiRes produces activity maps which present activity values at the single spot/cell level, and outputs the full activity matrix that comprises the activity values for every miRNA in every spot/cell. The user can use this matrix to perform further analysis.

Comment	Response
the heterogeneity of the single-cell sample.	
6. Additionally, averaging the scores on healthy and MS patients together is even less clear, and it may be better to remove this mode or change it to be only on the MS PBMCs.	We did this to demonstrate how the two different modes of miTEA-HiRes yield overlapping results in some scenarios, but offer distinct insights in others. In the breast cancer dataset, there was minimal overlap between the total and comparative mode lists, indicating that the miRNAs generally active in cancer were not specifically associated with the migratory vs. static condition. However, in the MS dataset, there was a significant overlap between the lists, suggesting that the miRNAs generally active in PBMCs and CSF are also the ones exhibiting differential activity between the MS and control groups.<br>It is true that in the MS case, the same biological conclusion could potentially be reached by first applying the total activity mode solely on the healthy samples and then comparing the resulting miRNA list to the one obtained by comparing MS to healthy in the comparative activity mode. However, this approach would involve using different datasets for the two modes, failing to demonstrate scenarios where miTEA-HiRes produces similar lists in both modes.
7. Line 190 – Are the selected 10,000 cells equally distributed among healthy and MS patients? If not, it might affect the results.	Yes, following your comment we added this detail explicitly (line 208):<br>“(5,000 from each group of MS and control)”
8. Figure 3: Some miRNAs show a significant difference in activity between control and MS samples. However, based on their p-values, they are also significant in each condition alone. What does it mean when there is a difference in activity between conditions if the miRNA is active in both conditions? Could it be due to different targets, resulting in different scores? If so, it would be interesting to investigate. Can you check for the targets that are	Very interesting question.<br>In order to answer it, we performed the following analysis: we focused on the nine miRNAs that appear at the top 10 list of MS PBMCs for the ‘total activity’ mode, as well as at the top 10 list of the ‘comparative’ mode for MS vs. control. We then performed normalization and z-scoring of the raw expression matrix (as explained in the paper). Then, for each cell, we ranked its genes based on their expression, and we computed the median rank of each gene in the MS and control populations. Finally, we computed the

Comment	Response
associated with each condition? Specifically, those that are enriched in the mGH test of each condition.	Spearman correlation coefficient between the vector of median ranks of the targets of a specific miRNA- in MS vs. control. We found that for the nine miRNAs mentioned, the correlation was high (above 0.91 for all), suggesting that while down-regulated targets were not significantly altered between MS and control, their degree of down-regulation was.<br>Nonetheless we kept investigating, now asking whether there were specific targets that have significantly different median ranks between MS and control. We found that there were few (for each of the nine miRNAs, no more than 12 targets with an absolute difference in median ranks of at least 200). Though statistical significance could not be achieved, many of the targets that were significantly more down-regulated in control cells than in MS cells were related to interferon alpha and gamma responses.<br><br>We are happy to add this analysis to the Discussion or to the Results should the reviewers/editors find that it adds to the paper. If requested to add this analysis we can also provide the corresponding gene lists and the associated graphics. This analysis can be added as a supplement.
9. Additionally, it is important to specify the number of cells from each population since, in some comparisons, it seems that the difference between populations is insignificant due to the number of cells and not due to lack of difference (for example, miR-519a-3p B1 population in the CSF data).	This is correct, thanks for pointing this out. We added the sample compositions as Supplementary Table 4. We also added compositions for the other datasets.
10. Table 3 – Why is the comparison only between 2 cell types? Why not compare the activity scores across all cell types? I would add a possibility to compare the activity scores between multiple groups.	Agreed. We will include support for multiple pairwise comparisons in the next version of miTEA-HiRes.
11. Figure 4: d-e seems significant	Yes, there are cases (such as this one) in

Comment	Response
between static and migratory cells only due to one dataset (SUM159). As a result, their histograms display a slight separation between static and migratory cells (h-i). To account for miRNAs that are significant in only one dataset, it will be worthwhile to analyze the effect of each dataset individually as well as collectively.	which a certain subpopulation may affect (here, slightly, as you point out) the comparative analysis results. On the one hand, grouping the datasets gives more statistical power. On the other hand, working on each subpopulation separately, assuming the subpopulation sizes allow it, gives more accurate conclusions (as we showed for the MS dataset). Here we focused on comparing static to migratory cells. The purpose of the miTEA-HiRes analysis is to point out phenomena. As in many high-throughput investigations, a more detailed follow up will be necessary in order to establish understanding of the real biology. In this case - is miR124 differentially active in migratory vs static in all breast cancer types or is this the case, for example, only in certain types of TNBCs? As we said before - preliminary directions can be inferred from deeper analysis, by the user, of the output matrix.
12. Section 2.5 –To rank the miRNAs by expression levels, the authors summed the raw counts of each miRNA across all the cells. This was likely done because single-cell data is often sparse. However, I'm not entirely sure why they chose to do this, as by averaging across all cells, we lose the variability of the single-cell data and mix signals from cells where the miRNA is active with cells where it is not. What is the advantage of performing this analysis over using bulk data?	Our purpose in evaluating totalRNA data was to understand the correlation between single-cell miRNA expression and activity, as a method validation approach. We did not find such correlation, perhaps due to data sparsity as you mentioned, or the reported sensitivity issue in the totalRNA technique, or the fact that expression and activity are related but are not expected to behave exactly the same. Observing the data, we could clearly see that when a miRNA is expressed even to a small extent it was also active, and when it was not expressed, it was also inactive. This is quantified by our mHG analysis, showing that the list of sorted expressed miRNAs is enriched with active miRNAs and also that non expressed miRNAs are enriched with inactive miRNAs. True - this is after averaging, for the reasons mentioned above. We note that previous work, including miTEA (Steinfeld, Israel, et al. "miRNA target enrichment analysis reveals directly active miRNAs in health and disease." *Nucleic acids research* (2013)), as well as Baek, Daehyun, et al. "The impact of microRNAs on protein output." *Nature* (2008) and Selbach, Matthias,

Comment	Response
	et al. "Widespread changes in protein synthesis induced by microRNAs." *nature* (2008), demonstrated the link of expression to activity, in the bulk context.<br>As no other tool has yet managed to demonstrate single cell expression to activity correlation, we believe this pseudo-bulk analysis does take us a step forward. Of course it would be interesting to investigate this on the single-cell and not pseudo-bulk level, when more mature single-cell mRNA-miRNA techniques are available.<br>Note that we have moved this section to be under the new 'Validation and comparison to other methods' (see Section 3.2).
13. Line 363 – If I understand correctly, you are using the data from HEK293T and MCF7 together as one sample, which introduces even more "noise" or heterogeneity. The same applies to the mouse data, where you are combining all stages of embryonic stem cell differentiation. Have you considered analyzing each dataset alone? It might lead to clearer insights.	Thank you for your comment. miTEA-HiRes uses z-scores and therefore requires a reasonable number of cells to perform optimally. As we scanned the literature to find sequencing methods that sequence both mRNAs and miRNAs to perform this validation, we found that the only method that was able to detect a sufficient amount of miRNA reads was Smart-seq-total. We used both datasets offered in their paper. However, we had to merge the human cell types, as well as the mouse development datasets, in order to gather enough cells to perform miTEA-hiRes's statistics reliably. Once methods of this sort mature, it would be valuable to perform this validation on bigger datasets (that offer both mRNA and miRNA reads), and separate between cell types or states as much as possible (in a way that still preserves statistical power).
14. Lines 407-414: This scenario can occur if the miRNA activity originates from a particular group within the sample with a strong signal that can influence the entire population. This highlights the importance of avoiding averaging activity scores across all cells but instead focusing on specific groups of cells with similar characteristics. Doing so helps to preserve the unique attributes of	Again - the comment regarding averaging is totally valid.<br>Summarizing earlier discussion, this is generally true, also for bulk data analysis. miTEA-HiRes, through the full matrix output, thus allows the user to perform differential analysis to any level of granularity, as long as there remains sufficient statistical power.<br>We will include some of your suggestions in future works, such as clustering the cells and multiple group comparisons. We feel that at

Comment	Response
individual cells in the dataset and avoids incorrect interpretations of the data.	this point such expansions of the functionality are out of scope.
Minor comments:<br>15. Figure 1: Change sample to cell, confusing.	We used “sample” since it can be either “cell” (single cell data) or “spot” (spatial data) or even bulk sample. We understand this might be confusing but cannot think of an alternative. We are open for suggestions. In certain places we did use spot/cell but this doesn't cover bulk, which we wanted to cover in Figure 1.
16. Figure 2: The label of the color bar is very small and hard to read, in addition, it should be -log10(activity p-value) and not p-value(-log10). The labels in other figures are also very small (for example, Figure 4).	Thanks. Fixed.
17. Figure 4 – In the text, it initially referred to figures 4h-k and later to figures 4f-g.	Thanks. Fixed.
18. Figure 5: Only appears in the discussion part, not referred to in section 2.5.	We removed this figure since it also appears as Supplementary Figure 7.
19. Table 1: Very hard to see the histograms of the p-values.	Thank you for bringing this to our attention. We have increased the size of the histograms in Table 1 and Supplementary Table 1 to enhance their visibility.
20. Why, for miR-19a-3p, is there no p-value and histogram for the comparative activity mode?	Since miR-19a-3p is not ranked in the top 10 of the comparative activity mode (same as miR-155-5p, which is significant in the comparative mode but not in the total activity mode).<br>We can add these two but think that it can be even more confusing.
21. Lines 356 – 357 – The author can check their assumption using pathway enrichment of the specific targets of these miRNAs which are lowly expressed in this sample.	Thank you for this great suggestion. We implemented it (see lines 380-386, and Supplementary Fig. 5).

Comment	Response
Reviewer #3	
Herbst et al. present an interesting approach to look at miRNA activity in single cell and spatial datasets, relying on the expression patterns of targets (with miRNA-target interaction supported by experimental evidence). The method is exemplified in multiple spatial and single cell transcriptomics datasets and results seem to match literature or provide novel insights for less studied miRNAs. By providing a github repository and pip install, the authors also facilitate other researchers to apply their approach to novel datasets.	We thank the reviewer for the very careful reading of the manuscript and the special attention to the details of the statistics. We greatly appreciate the review and the enlightening comments.
I have a few comments, mainly regarding the method:<br>1. Line 498-511: Are differences in coverage taken into account during the preprocessing steps (e.g. by downsampling or by requiring a minimum number of reads)? In case there are both cells with few reads and cells with considerably more reads, the counts of (the fewer) detected genes in the first may be inflated after normalization (which will impact the z-scores per gene).	We agree that including cells with very few reads may result in magnified z-scores in these cells, potentially further affecting the following miRNA activity analysis. We therefore added a recommendation for the user to pre-filter the data before applying miTEA-HiRes (lines 118-120), and we will include this step in next versions of miTEA-HiRes. We also looked into the single-cell datasets included in this manuscript to make sure this did not affect our results. The breast cancer dataset was already pre-filtered, so all cells had at least 1000 reads.<br><br>The added text in lines 118-120: “We recommend preprocessing data to account for batch effects if relevant, and to remove cells with very few reads (<1000) before using it as input for miTEA-HiRes, as these cells may disproportionately impact z-scores.”
2. The activity p-values are currently corrected for multiple testing, yet there are many tests performed in one dataset analysis. The provided p-values may as such be overoptimistic and a multiple testing correction or at least a remark may be appropriate. For the comparitive activity mode scores, the authors do use FDR correction and they also	A very valid point. Thank you.<br>In the MS dataset we do perform multiple comparative analyses, as there are many cell types and about 100 possible pairwise comparisons (we can compare every pair of cell types within the MS or healthy samples, as well as compare MS vs. healthy for each cell type). Bonferroni correction for this will not significantly affect most of the presented significant p-values (see Tables 2 and 3).

Comment	Response
prioritize smaller activity p-values later in the paper.	However, as this comment is important, we took the reviewers recommendation and added text in lines 277-282.<br>The added text: “We note that while the presented WRS p-values are corrected for the number of evaluated miRNAs, they are not adjusted for multiple pairwise comparisons, for instance, between all pairs of cell types. Users can correct their p-values for multiple comparisons by multiplying by the number of comparisons. For the MS dataset, even if all relevant pairwise comparisons are performed (~ 100), this correction would not affect conclusions regarding significant patterns (colored in pink in Tables 2 and 3).“
3. The activity score calculation is not entirely clear to me. For the single cell data, this is an adjusted p-value (average or based on Wilcoxon rank-sum test) where a smaller activity score indicates miRNA activity. For spatial data, however, it is explained as the percentage of spots that has an activity p-value below a certain threshold. In this case, a higher activity score seems to indicate miRNA activity. Is this correct? Can this be further explained in the methods section?	Yes, this is correct.<br>For spatial data sets, we chose to order the miRNAs based on the percentage of active spots in the sample (in descending order), since we thought this is the best measure of activity across an entire slide. This measure is not a significance score and therefore cannot be corrected for multiple testing.<br>For single cell datasets in total activity mode, we chose to order the miRNAs based on their average activity p-value over all cells, which we then corrected for multiple testing.<br>For single cell datasets in comparative activity mode, we order the miRNAs based on their Wilcoxon rank-sum p-value for the comparison between the two groups, which we then corrected for multiple testing.<br>We reviewed the explanations in the text to make sure they are as clear as possible (Results- Section 2.1, and Methods- Section 5.1.5).
4. On line 222, the authors deviate from the default of 10k cells. How was the number of cells in general determined and can the authors provide guidelines for other users about the optimal number of cells for a particular analysis?	The default 10k cells is a good pick since it reduces computation time, but also is sufficient to maintain statistical power, both for the total activity mode (where activity scores are averaged across all 10k cells) and for the first application of the comparative activity mode, where we compared 5000 MS cells vs. 5000 control cells.<br>However, we then investigate cell type specific miRNA differential activity. We know

Comment	Response
	that some cell types are more abundant and others are more scarce. We wanted to make sure that we would have sufficiently many cells from every cell type to support meaningful statistical evaluation of the difference in miRNA activity in every pair of cell types (and also, compare the same cell type in MS and control). We therefore used more cells for this analysis.<br>We added a guideline in lines 642-643. Added text: “We recommend using the comparative activity mode to compare populations of at least 100 cells in each.”
5. Supplementary Fig. 4 is not entirely clear for me. A & b are from one dataset, c&d from another, with black lines representing either the active or non-active miRNAs. Taking into account the different ranking (descending in a&c, ascending in b & d) and thus mirroring effect, the black bars still do not replace all white space and vice versa when comparing a&b or c&d (active vs inactive miRNAs). Should this not be the case?	Thanks for this comment. The analysis is a bit complex. There is no mirror effect due to the fact that some miRNAs are neither “active” or “non-active” (but rather, something in the middle or cannot be determined). We added a link in the figure description/caption to Methods section 5.6 (where this is explained).<br>In short:<br>A miRNA was considered active if its activity score was less than 0.05 (average activity p-value over all cells). miTEA-HiRes' MTI database distinguishes between miRNAs with 3p and 5p strands, while the total-RNA dataset displays only one value for the two strands, which represents the summed expression of them. To account for this, we considered a miRNA to be active if at least one of the strands was active.<br>A miRNA was considered to be non-active if its activity score was greater than 0.1 (average activity p-value over all cells). If a miRNA was found to be non-active, but its other strand (3p or 5p) was previously considered as active, it was excluded from the non-active miRNAs list to ensure that only truly non-active miRNAs were included.
Minor comment:<br>6. Some typos in the Github readme (e.g. comPArative activity, traNscriptomics, compuTation)	Thank you! We fixed the typos.

Comment	Response
Reviewer #1	
My previous concerns were addressed in this revision. The manuscript reads much more clearly to me. With more understanding, I have the following comments.	We thank the reviewer for the well appointed comments, following which the manuscript now incorporates visuals of different angles, providing the reader with more understanding about the method and the underlying biological pattern.
1. P-values are compared using Wilcoxon rank sum test in the “comparative activity mode”. But I think the “effect sizes” is usually the focus of comparisons, I.e., given two groups (A and B) and a miRNA, the most direct question is whether the target genes in group A are of lower expression than those target genes in group B. Does Wilcoxon rank sum test of p-values lead to the same conclusion as using Wilcoxon rank sum test on the target genes between the two groups? Or can this point be at least discussed?	We have added boxplots of miR-519a-3p and miR-651-3p target expression for MS Vs. control, corresponding to Figure 3, as well as WRS p-values. See Supplementary Figure 11, and also lines 152-153, 272-280, and 542-544. We believe this visualization displays the biological effect clearly and we thank you for the suggestion.
2. Another question about p-values is that they are averaged in the “total activity mode”. But there are many other ways to combine p-values, such as Fisher's method (which derives the expected distribution when summing up the log of p-values) or Cauchy combination rule. Why using average rather than other p-value aggregation methods? And why averaging p-values rather than log-transformed p-values?	This point is discussed in lines 654-655 under the methods section, and we have added references to Fisher's method and Cauchy combination rule as per your suggestion. We are planning to incorporate other aggregation methods in future versions. However, in the meantime, the users are provided with the activity p-value matrix per cell, and they may apply other aggregation methods as downstream analysis steps.
3. Following the comment of reviewer 2 in the previous review, the statement “For example, the activity of miR-29c-3p in the breast cancer sample (Fig. 2, second row) does not precisely correspond, at the qualitative visual inspection level, to either the clustering patterns or the characteristics observed in the H&E	We have included your suggested experiment and downsampled miR-16-5p target genes, as well as all genes (in two separate analyses). The produced activity maps are similar to the original one, and are not blurry as for miR-29c-3p. Nonetheless, we believe this is an important concern and we were happy to address it in the paper. See Supplementary Figure 12 as well as lines

Comment	Response
slide" in line 175—177 is somewhat suspicious in my view, as the activity map looks like a blurry version of that of miR-16-5p. As spatial Transcriptomics data is sparse and the ranking of gene expression may be affected by random variation, I wonder if the target genes of a miRNA in general have low expression and a high percentage of zeros across the spots, can the activity map be accurately identified? A potential experiment is to down-sample the counts of target genes related to miR-16-5p (e.g. each UMI has 0.5 probability to retained or set to 0 for each spot for each target gene) and infer the activity map, whether the activity map is visually consistent with the original one before down-sampling.	179-181.
4. The notation Λ in equation (1) after line 624 doesn't seem to be defined.	Thank you for bringing this to our attention. A definition was added in lines 639-640.
Reviewer #2	
In this revision, the authors have primarily made changes to the text rather than introducing new analyses. While this approach is acceptable in most instances, there are specific cases where incorporating the requested analyses would significantly enhance the quality of the paper and its conclusions.	We thank the reviewer for the additional analysis approaches, which we performed and believe indeed enhance the quality of the paper.
1. For Figure 2, a straightforward analysis of adding a boxplot of the p-values activity scores of each spot in the different GE clusters. This would help evaluate whether a specific miRNA activity is associated with a particular cluster.	Following your feedback, we have added a boxplot of the activity p-value distribution for each of the GE clusters in supplementary figure 10 with reference from line 179.
2. I am still unclear about the meaning of the miRNAs that show differing activity between control and MS samples	That is an interesting analysis approach and we have added a boxplot of miR-519a-3p and miR-651-3p target expression for MS Vs.

Comment	Response
(Figure 3), while they are also significant in each condition alone. Further clarification is needed. I understand that you could not achieve statistical significance, but it would be beneficial to display the expression levels of the target genes in both control and MS cells to better understand this difference. Is there a variation in target expression between the two groups?	control, corresponding to Figure 3, as well as WRS p-values. See Supplementary Figure 11, and also lines 152-153, 272-280, and 542-544. We believe this visualization displays the biological effect clearly and we thank you for the suggestion.
Reviewer #3	
The authors have carefully considered my comments from the previous review and have successfully addressed them in the revised manuscript. In particular, they have provided clarifications and added appropriate warnings in the text where necessary.	Thank you!
